# Cooperative duplex formation by synthetic H-bonding oligomers†
†Electronic supplementary information (ESI) available: Detailed experimental procedures with spectroscopic characterization data, ^31^P and ^1^H NMR titration spectra, binding isotherms, limiting chemical shifts for free and bound states, and thermal denaturation spectra. See DOI: 10.1039/c5sc03414k


**DOI:** 10.1039/c5sc03414k

**Published:** 2015-10-22

**Authors:** Alexander E. Stross, Giulia Iadevaia, Christopher A. Hunter

**Affiliations:** a Department of Chemistry , University of Cambridge , Lensfield Road , Cambridge CB2 1EW , UK . Email: herchelsmith.orgchem@ch.cam.ac.uk

## Abstract

A series of flexible oligomers equipped with phenol H-bond donors and phosphine oxide H-bond acceptors have been synthesised using reductive amination chemistry. H-bonding interactions between complementary oligomers leads to the formation of double-stranded complexes which were characterised using NMR titrations and thermal denaturation experiments. The stability of the duplex increases by one order of magnitude for every H-bonding group added to the chain. Similarly, the enthalpy change for duplex assembly and the melting temperature for duplex denaturation both increase with increasing chain length. These observations indicate that H-bond formation along the oligomers is cooperative despite the flexible backbone, and the effective molarity for intramolecular H-bond formation (14 mM) is sufficient to propagate the formation of longer duplexes using this approach. The product *K* EM, which is used to quantify chelate cooperativity is 5, which means that each H-bond is more than 80% populated in the assembled duplex. The modular design of these oligomers represents a general strategy for the design of synthetic information molecules that could potentially encode and replicate chemical information in the same way as nucleic acids.

## Abstract 2

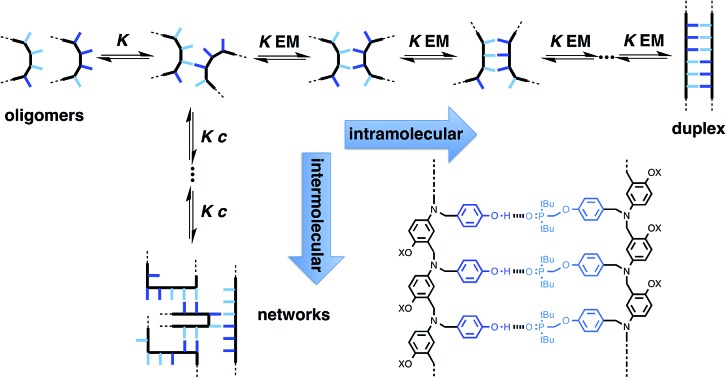
Flexible phenol-phosphine oxide oligomers show promise as a new class of synthetic information molecule.

## Introduction

Formation of intermolecular complexes between two linear polymers equipped with complementary recognition sites is the molecular basis for life on Earth. As pointed out by Watson and Crick in 1953, this supramolecular architecture provides a robust mechanism for encoding molecular information and for replication of this information through template-directed synthesis.^1^ Although synthetic polymers bearing side-chain recognition sites have been reported, these systems are generally of ill-defined chemical and supramolecular structure and lack the control over length and sequence found in biological polymers.^2^ Stepwise synthesis of short oligomers provides access to more well-defined systems, and a number of synthetic supramolecular systems that form duplex structures have been reported.^3^ However, the recognition sites in these compounds are usually integrated into the backbone of the oligomer, which limits their versatility because a rather precise matching of molecular geometries is required. In contrast, the modular architecture of nucleic acids appears to be remarkably robust with respect to chemical manipulation.^4^ Re-engineering experiments on nucleic acids show that it is possible to substitute the sugar,^5^ the phosphate,^6^,^7^ or the bases,^8^ for very different chemical components and still retain the duplex forming properties of the system. These results suggest that attempts to develop new synthetic information molecules would benefit from a modular strategy that would allow independent optimisation of the different components of the monomer units.^9^ This report describes the first steps towards such a system with the synthesis of complementary oligomers that form stable duplexes in organic solvents.

## Approach

**Fig. 1:**
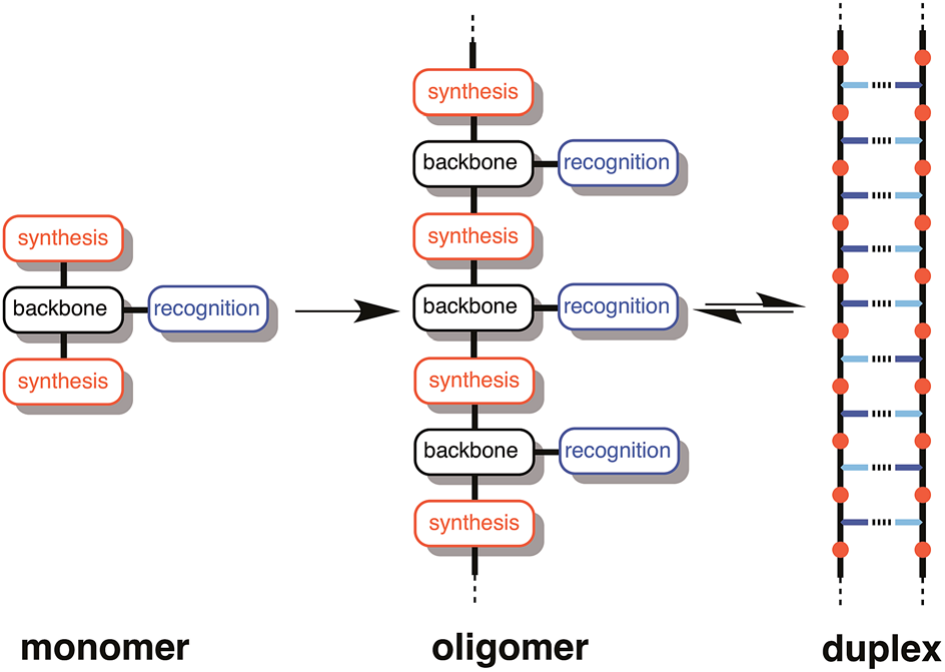
Blueprint for a synthetic information molecule. The key design components are the covalent chemistry used for synthesis (red), the non-covalent chemistry used for recognition (blue), and the backbone linker that determines the geometric complementarity of the two chains (black).

Fig. 1 illustrates the DNA molecule stripped down to the basic constituents required for duplex assembly and template-directed synthesis: a recognition-based pairing system (blue), chemistry for the synthesis of oligomers (red), and a linker that allows the recognition sites on the two polymeric backbones to reach one another (black). This basic blueprint provides guidelines for the construction of a wide range of different types of molecule that could function in the same way as nucleic acids under appropriate conditions. Nucleic acids operate in water, where stacking of the hydrophobic bases is the main driving force for duplex assembly.^10^ This supramolecular architecture requires mutual complementarity of the chemistry, linker and recognition elements, so that the duplex is tightly packed. Here, we target synthetic duplexes held together by H-bonding interactions in organic solvents, so that a much looser structure is possible with fewer constraints on the components. The modular design in Fig. 1 will allow for the future optimization of the properties of these components independently.

**Fig. 2:**
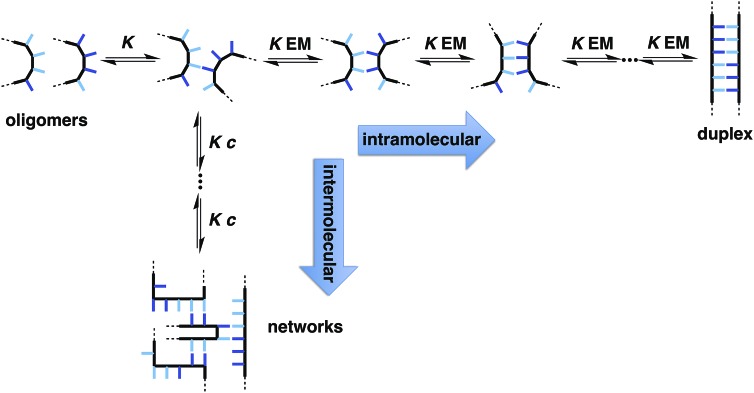
Recognition-directed assembly of a duplex between two complementary oligomers (intramolecular channel) competes with uncontrolled assembly of supramolecular networks (intermolecular channel). *K* is the association constant for formation of an intermolecular interaction between two recognition elements (blue bars), EM is the effective molarity for formation of an intramolecular interaction in the duplex structure, and *c* is the operating concentration. For specific oligomers, the equilibrium constants have additional statistical factors reflecting the differences in the degeneracy of the complexes.

The two key parameters that determine the efficiency of duplex formation between two oligomers are the association constant for the intermolecular interaction between two recognition sites (*K*) and the effective molarity for formation of the corresponding intramolecular interactions as the duplex zips up (EM).^11^Fig. 2 shows the steps involved in the assembly of a duplex from two complementary oligomers. The first step is an intermolecular interaction with an association constant *K*. Formation of subsequent interactions on the intramolecular pathway leading to duplex formation requires that the product *K* EM is greater than unity. There is a competing intermolecular assembly channel, and the ratio of EM to the operating concentration (*c*) determines whether uncontrolled interactions between multiple partners leads to aggregation and formation of cross-linked supramolecular networks rather than a well-defined duplex. Thus the criteria for high fidelity duplex formation are *K* EM ≫ 1 and EM ≫ *c*.

Qualitatively, EM increases with geometric complementarity and decreases with conformational flexibility, but it is still difficult to make quantitative predictions of the value of EM as a function of chemical and supramolecular structure. We have shown that for supramolecular architectures of varying complementarity and flexibility, the values of EM for intramolecular interactions fall in a surprisingly narrow window (10–1000 mM)^12^ compared with covalent EMs, where changes in chemical structure can lead to variations of several orders magnitude.^13^ Although there is a trade-off between conformational flexibility and geometric complementarity, the margin for error in supramolecular design is much higher if more flexible molecules are used, and the associated decrease in EM is not dramatic.^14^ The consequence is that if *K* ≫ 100 M^–1^ for the recognition modules in Fig. 1, it should be possible to assemble duplex structures without the need for careful design of the linker module, provided the backbones are sufficiently flexible to ensure geometric complementarity.

**Fig. 3:**
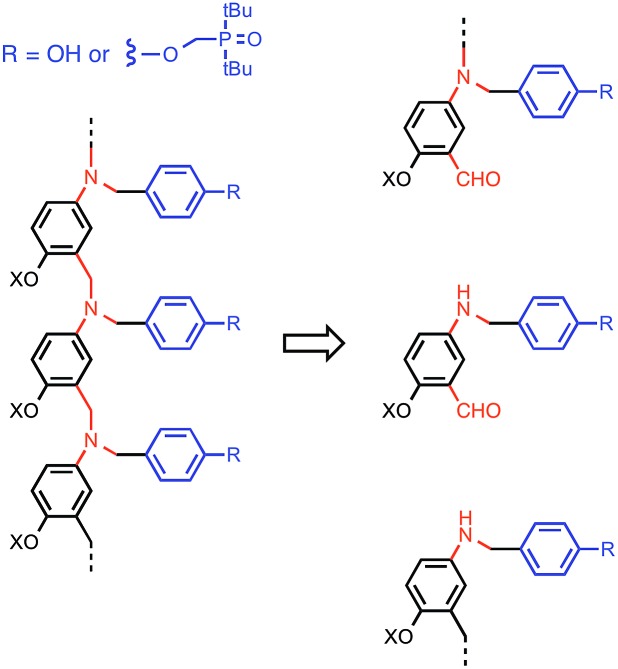
Design of a synthetic information molecule (*cf.*Fig. 1), which can be synthesised by reductive amination of aminoaldehyde monomers (red) equipped with a phenol or a phosphine oxide recognition module (blue). X is a site for attachment of solubilising groups.

This *K* criterion dictates the choice of recognition modules. The simplest possible recognition system is a single H-bond between a H-bond donor (D) and a H-bond acceptor (A), *i.e.* a two-letter alphabet rather than the four-letter alphabet used in nucleic acids. Provided the backbone does not have any polar functional groups that could compete for the H-bonding interactions, use of simple H-bonding modules will ensure selectivity of the recognition-based pairing system: D will only pair with A; D will not pair with D, and A will not pair with A. Phosphine oxides and phenols form exceptionally stable H-bonds in toluene (10^2^ to 10^3^ M^–1^),14*g* and so these functional groups were chosen for the recognition modules in the oligomer design shown in Fig. 3. The backbone of the oligomer in Fig. 3 contains no H-bond donors, and the backbone aniline nitrogen and aromatic ether oxygen atoms are both weak H-bond acceptors (the H-bond acceptor parameter *β* ≈ 4 and 3 respectively) compared with phosphine oxide (*β* ≈ 10).^15^ Competition of the backbone with the recognition sites will therefore be negligible. The ether oxygen provides a convenient point for addition of side-chains to control the solubility of the oligomers (X in Fig. 3). Here we report the synthesis of phenol and phosphine oxide oligomers from the corresponding aminoaldehyde monomers and NMR experiments to characterise assembly of the corresponding duplexes.

## Results and discussion

### Synthesis

**Scheme 1:**
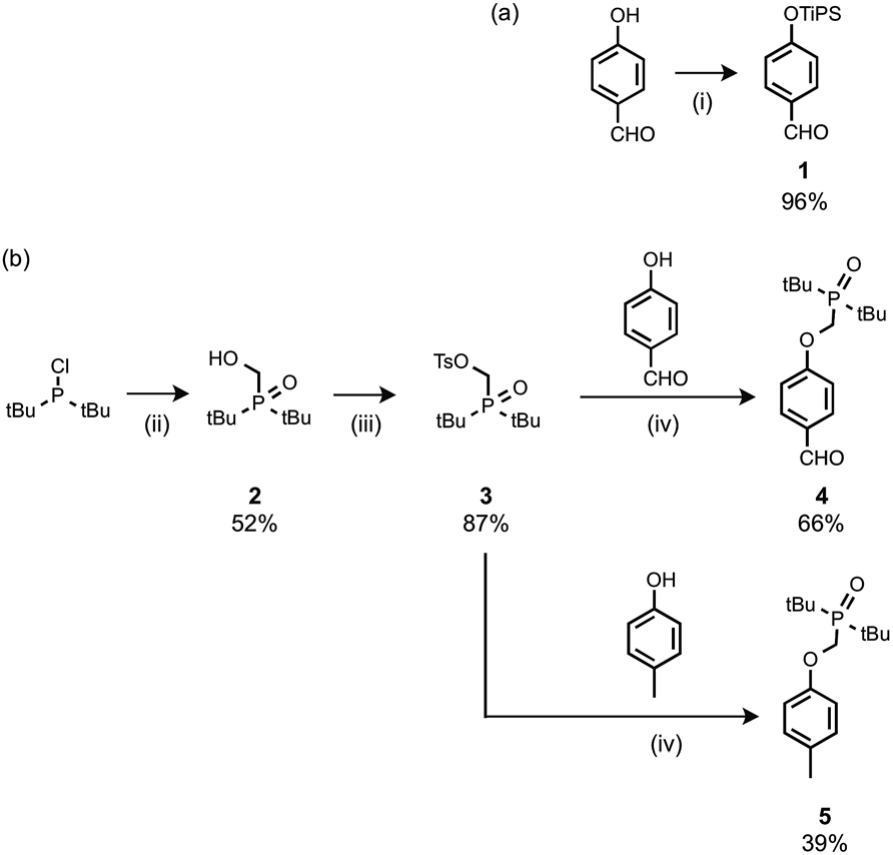
(i) Tri-*i*-propylsilyl chloride, imidazole; (ii) formaldehyde, aq. HCl; (iii) *p*-toluenesulfonyl chloride, NEt_3_; (iv) Cs_2_CO_3_.

The recognition modules were derived from *p*-hydroxybenzaldehyde (Scheme 1). The phenol hydroxyl group was protected as the silyl ether **1** for the synthesis of oligomers.^16^ Oxidation of di-*t*-butylchlorophosphine in the presence of formaldehyde gave **2**, which was treated with *p*-toluenesulfonyl chloride to give **3**.14*g* Alkylation of *p*-hydroxybenzaldehyde with **3** gave the phosphine oxide H-bond acceptor unit, **4**. Phosphine oxide **5** was prepared by alkylation of *p*-cresol with **3** (Scheme 1b). **5** is a single H-bond acceptor compound (A), which was used with *p*-cresol, the corresponding single H-bond donor compound (D), for determining the strength of an intermolecular phenol–phosphine oxide H-bond.

**Scheme 2:**
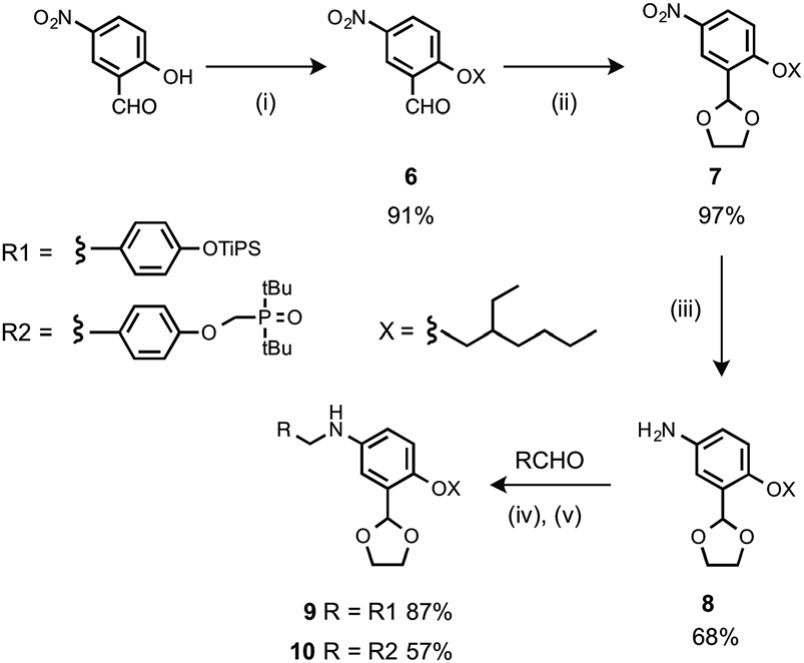
(i) 2-Ethylhexyl bromide, K_2_CO_3_; (ii) *p*-toluenesulfonic acid, ethylene glycol; (iii) H_2_, Pd/C; (iv) heat; (v) NaBH_4_.

The monomer units for oligomer synthesis were prepared according to Scheme 2. 2-Hydroxy-4-nitrobenzaldehyde was alkylated with 2-ethylhexyl bromide to give **6**, which was protected as the corresponding acetal, **7**, by condensation with ethylene glycol. Reduction of **7** gave the primary aniline **8**. Formation of the corresponding imines using **1** or **4** and reduction with sodium borohydride gave the required monomers **9** and **10**.

**Scheme 3:**
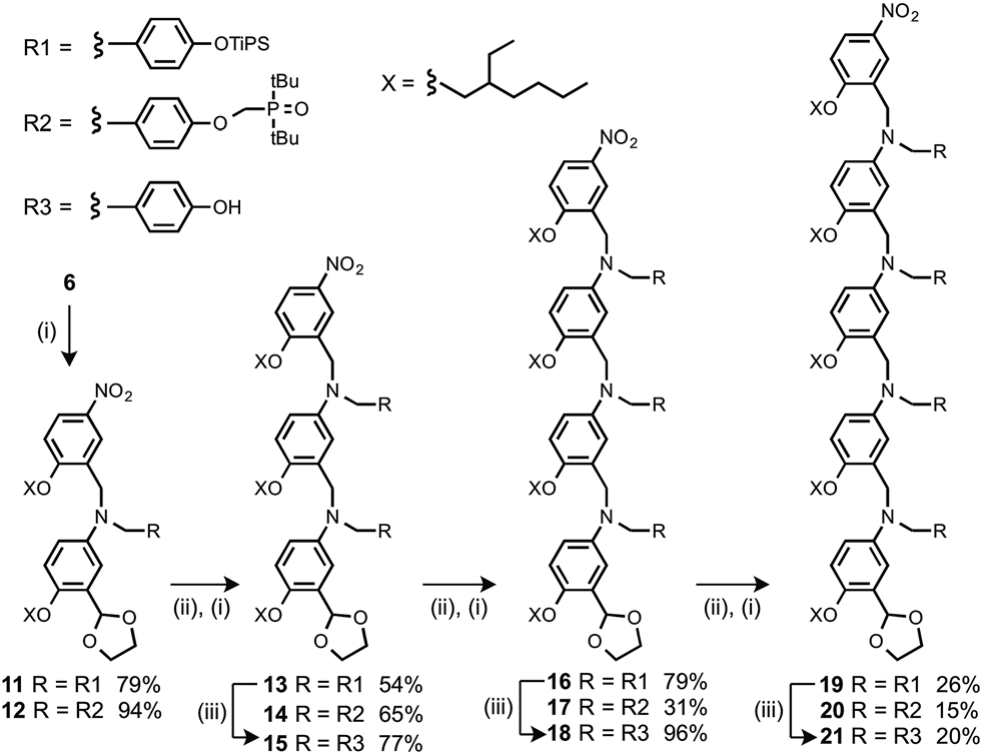
(i) **9** or **10**, NaBH(OAc)_3_; (ii) aq. HCl; (iii) TBAF.

Oligomers were prepared by building a chain starting from **6** (Scheme 3). Reductive amination of **6** with the monomer units (**9** or **10**) using NaBH(OAc)_3_ gave the 1-mer chains, **11** and **12**.^17^ The acetal groups were then deprotected using aqueous HCl, and the resulting aldehydes were coupled with a further monomer unit to give the 2-mers, **13** and **14**. **14** is the double H-bond acceptor AA, and deprotection of **13** using tetra-*n*-butylammonium fluoride (TBAF) gave **15**, the corresponding double H-bond donor DD. The same deprotection-coupling sequence shown in Scheme 3 was used to prepare the 3-mers, **17** (AAA) and **18** (DDD), and the 4-mers, **20** (AAAA) and **21** (DDDD).

### Binding studies

**Fig. 4:**
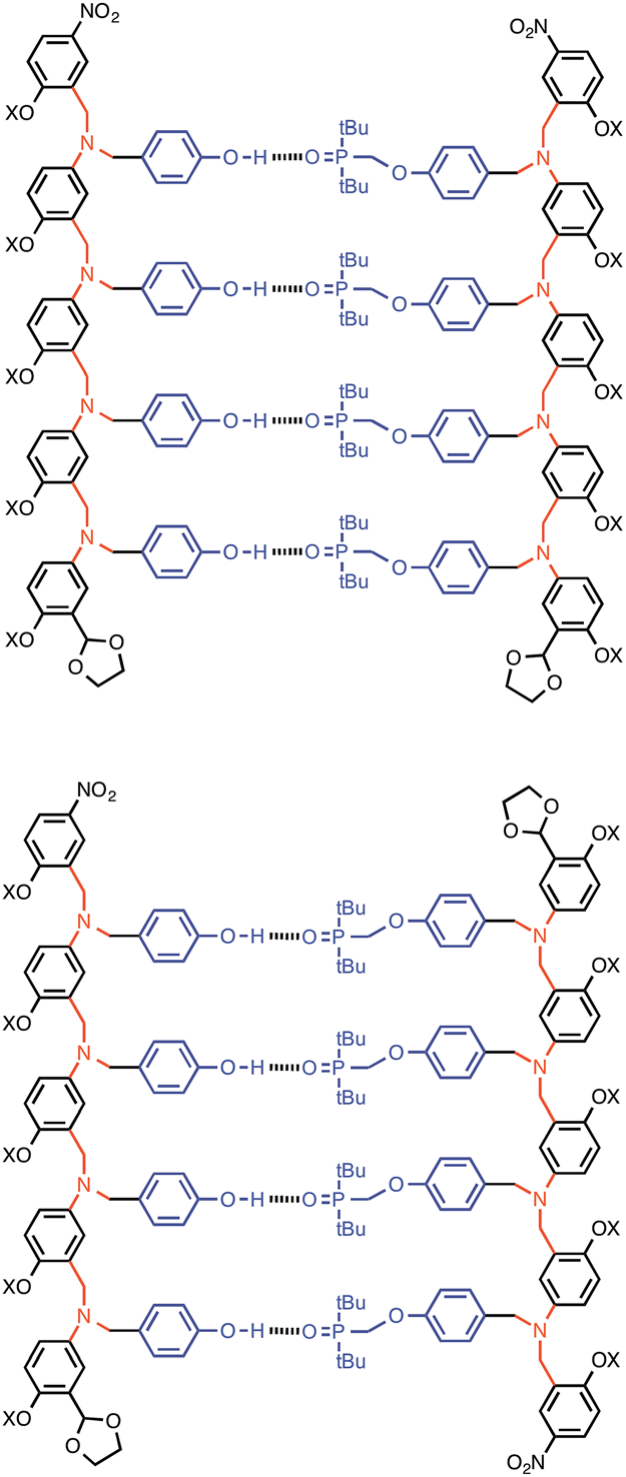
The AAAA·DDDD duplex structure: parallel or antiparallel structures are possible.

Complexation of length-complementary oligomers was studied using ^1^H and ^31^P NMR titration experiments in toluene. The chemical shifts of the ^31^P and ^1^H NMR signals observed for the oligomers are similar to the values observed for the monomer units, which indicates that there is no significant folding of the free oligomers and that the H-bonding sites are displayed in an accessible environment on the flexible backbone. The ^1^H NMR spectra of the oligomers are complicated with many overlapping signals, due to the large number of chemically similar protons. However, the ^31^P NMR spectra are much simpler, and large changes in chemical shift were observed on titration of the donor oligomers (D_*N*_, *N* = 1–4) into the acceptor oligomers (A_*N*_). All titration data fit well to 1 : 1 binding isotherms, and the association constants and limiting changes in ^31^P NMR chemical shift are reported in Table 1 (see ESI† for details). The complexation-induced changes in ^31^P NMR chemical shift are similar for all of the signals in all of the complexes (+4–5 ppm). A large positive change in ^31^P NMR chemical shift is indicative of H-bond formation^18^ and the results in Table 1 imply that all of the phosphine oxide groups are fully bound in all of the complexes. In other words, fully H-bonded duplex structures are formed (Fig. 4). Although the ^1^H NMR data are more difficult to interpret, the patterns of complexation-induced changes in chemical shift are also similar for all of the oligomers, which suggests that the duplexes have similar structures (see ESI†). The oligomer chains have directionality, so there are two possible structures for the duplexes: parallel and antiparallel (Fig. 4). The NMR data do not allow us to determine whether one of these is preferred over the other.

**Table 1:** Association constants (*K*_*N*_), effective molarities (EM) and limiting complexation-induced changes in ^31^P NMR chemical shift for formation of duplexes in toluene at 298 K^*a*^

Complex	log *K*_*N*_/M^–1^	EM/mM	*K* EM	Δ*δ*/ppm
A·D	2.5 ± 0.1	—	—	4.9
AA·DD	3.3 ± 0.1	8 ± 3	3 ± 1	5.3, 5.3
AAA·DDD	4.2 ± 0.1	14 ± 1	5 ± 1	4.8, 4.9, 5.1
AAAA·DDDD	5.4 ± 0.5	21 ± 8	7 ± 1	3.8, 5.1, 5.1, 5.1

^*a*^Each titration was repeated at least twice, and the average values are reported with errors (in brackets) at the 95% confidence limit.

**Fig. 5:**
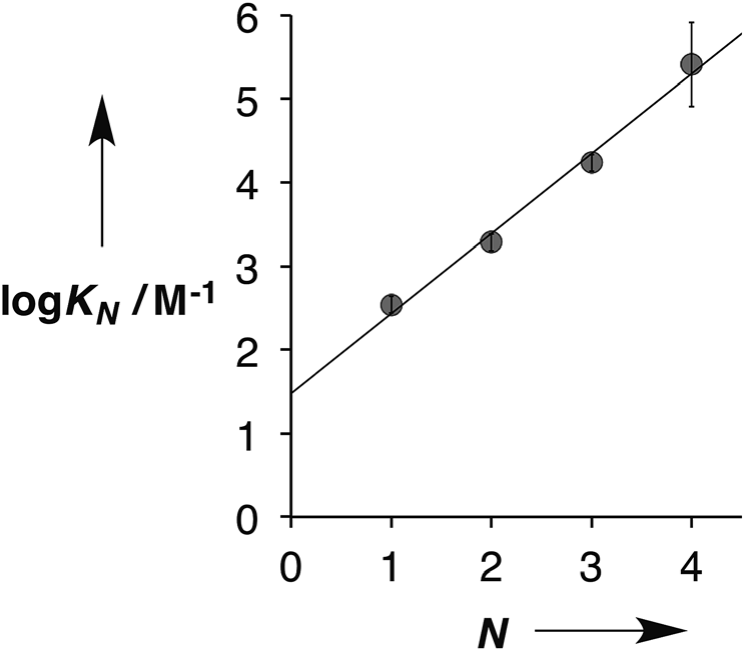
Relationship between the association constants for duplex formation in toluene at 298 K (*K*_*N*_) and the number of recognition modules in the oligomer (*N*). The best fit straight line is shown (*r*^2^ = 0.99).

Fig. 5 shows the association constants for duplex formation (*K*_*N*_) plotted as a function of the number of H-bonding sites *N*: the data fit well to a straight line, and there is a uniform increase in association constant of an order of magnitude for each additional H-bond formed (eqn (1)).

1 log *K*_*N*_ = 1.0*N* + 1.5

This relationship implies that the flexible backbone is able to adopt an ideal geometry for duplex assembly, so that the effective molarity (EM) for sequential H-bond formation in zipping up the duplex is constant. The association constant for duplex formation between two oligomers with *N* interaction sites (*K*_*N*_) can therefore be expressed as the product of stepwise equilibrium constants (*cf.*Fig. 2) for assembly of the duplex using a single value of EM (eqn (2)).

2 *K*_*N*_ = 2*K*^*N*^EM^*N*–1^

where the statistical factor of 2 assumes that the parallel and antiparallel structures in Fig. 4 are equally populated.

This relationship was used to calculate the values of EM in Table 1. Using the A·D association constant as *K* in eqn (2), gives an average value of 14 mM for the EM for duplex assembly. The chelate cooperativity associated with duplex assembly is quantified by the product *K* EM, and the data in Table 1 give an average value of 5. This value implies that duplex assembly is cooperative and that it should be possible to propagate the assembly of longer structures using this architecture. However, each H-bond is about 80% bound on average, so the duplex structures are dynamic with some fraying of the interaction sites.

### Thermal denaturation experiments

**Fig. 6:**
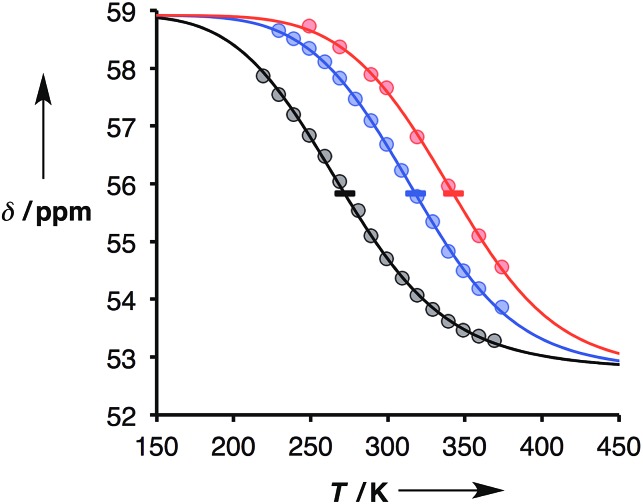
Experimental ^31^P NMR chemical shift plotted as a function of temperature for 1 : 1 mixtures (1 mM) of A·D (black), AA·DD (blue), and AAA·DDD (red) in toluene. The lines are the best fit to eqn (9) (total rmsd < 0.2 ppm), and the horizontal bars show the transition melting temperatures, *T*_m,*N*_.

Thermal denaturation data were measured by making 1 : 1 solutions of length-complementary oligomers at 1 mM concentrations in toluene and measuring ^31^P NMR spectra at temperatures from 228 to 373 K. At low temperatures, the ^31^P NMR signals moved to higher chemical shift, indicative of an increase in the extent of H-bond formation (Fig. 6).^18^ At higher temperatures, a decrease in ^31^P NMR chemical shift was observed, indicative of disruption of the H-bonding interactions. When a sample of phosphine oxide **5** was monitored over the same temperature range in the absence of a H-bond donor, the variation in chemical shift (≈1 ppm) was small relative to the changes observed for the H-bonded complexes (>4 ppm), confirming that the temperature dependence of the ^31^P spectra in Fig. 6 is related to duplex assembly and denaturation. The signals in the ^31^P NMR spectra of the AAAA·DDDD duplex become very broad and overlapped with changes in temperature, so it was not possible to extract an accurate melting profile for this system. However, the data for AAAA·DDDD are qualitatively consistent with a more stable duplex that melts at higher temperatures than the other three complexes (see ESI†).

The thermal denaturation experiment can be used to extract thermodynamic parameters for duplex formation by fitting the data to a two-state model,^19^ which assumes only duplex and single strands are present. At a given temperature *T*, the equilibrium constant for formation of a duplex between two oligomers with *N* binding sites, *K*_*N*_(*T*), is given by eqn (3).3
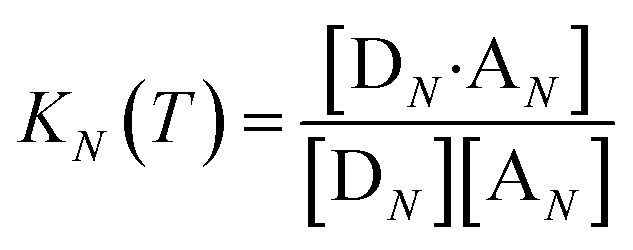
where [D_*N*_] and [A_*N*_] are the concentrations of single strand oligomers bearing donor groups and acceptor H-bonding groups respectively, and [D_*N*_·A_*N*_] is concentration of the duplex.

If the two oligomers are present in equal concentrations, the total concentration of all free strands can be written as *c* and the total fraction of all bound species as *α* (eqn (4)).4
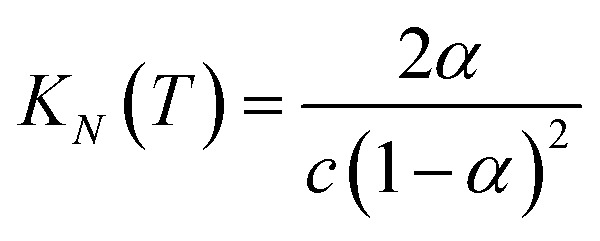



Rearranging eqn (4) gives an expression for *α* in terms of *K*_*N*_(*T*) and *c* (eqn (5)).5
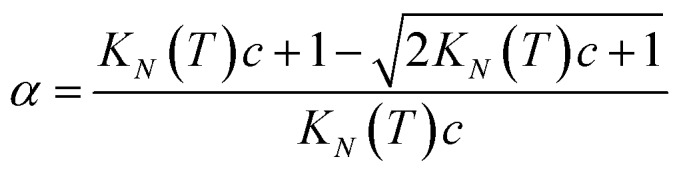



The observed chemical shift for a two state system in fast exchange, *δ*, can be expressed in terms of *α* by eqn (6).

6 *δ* = *δ*_f_ + (*δ*_b_ – *δ*_f_)*α*

where *δ*_b_ and *δ*_f_ are the chemical shifts of the duplex and single strand states.

Assuming that the enthalpy and entropy of duplex formation are temperature independent, and the change in heat capacity between free and bound states is zero, the temperature dependence of *K*_*N*_(*T*) can be analysed using the integrated form of the van't Hoff equation (eqn (7)).^19^7
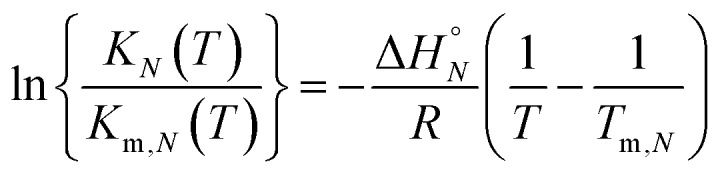
where *K*_*N*_(*T*_m,*N*_) is the association constant for formation of the D_*N*_·A_*N*_ duplex at the transition melting temperature, *T*_m,*N*_, Δ*H*°*N* is the enthalpy change for formation of the D_*N*_·A_*N*_ duplex, and *R* is the gas constant.

At the transition melting temperature *α* = 0.5, and using this value in eqn (4) gives *K*_*N*_(*T*_m,*N*_) = 4/*c*. Substituting for *K*_*N*_(*T*_m,*N*_) in eqn (7) and rearranging gives eqn (8), the temperature dependence of the association constant *K*_*N*_(*T*).8
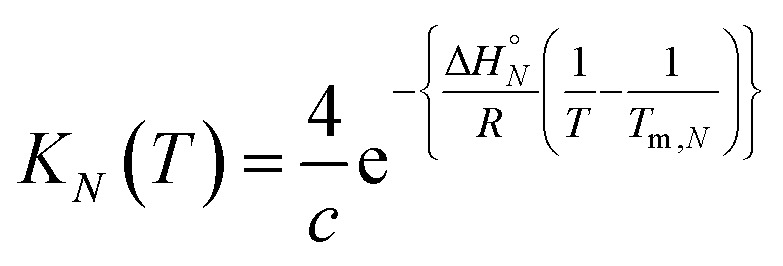



Combining eqn (5), (6) and (8), allows us to express the observed chemical shift in terms of *T*.9
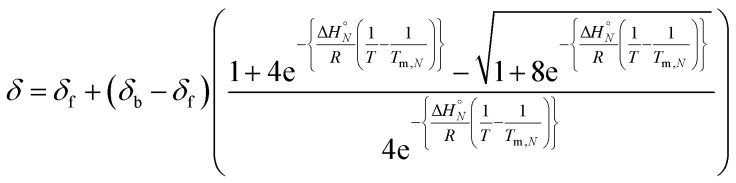



The thermal denaturation data for duplexes A·D, AA·DD and AAA·DDD were analysed by optimising the values of *δ*_b_, *δ*_f_, Δ*H*°*N* and *T*_m,*N*_ to minimise the difference between the experimentally measured values of *δ* and the values calculated using eqn (9). The values of *δ*_b_ and *δ*_f_ could depend on *N*, but the limiting chemical shifts determined by fitting the titration data recorded at 298 K are similar for all four duplexes (*δ*_f_ = 53.6–53.9 ppm, and *δ*_b_ = 57.9–59.0 ppm, see ESI†). We therefore fit the thermal denaturation data using the same value of *δ*_b_ and *δ*_f_ for all of the duplexes, and the results (*δ*_f_ = 52.8 ppm, and *δ*_b_ = 58.9 ppm) are consistent with the titration data. Fig. 6 shows the lines of best fit calculated using eqn (9) (solid lines) for each thermal denaturation data set, and Table 2 shows the values of the fitted parameters along with the values of Δ*S*°*N* and Δ*G*°*N* calculated from log *K*_*N*_ at 298 K.

**Table 2:** Thermodynamic parameters for formation of H-bonded duplexes in toluene determined using ^31^P NMR thermal denaturation data

*N*	*T*_m,*N*_/K	Δ*H*°*N*/kJ mol^–1^	*T*Δ*S*°*N* (298)/kJ mol^–1^	Δ*G*°*N* (298)/kJ mol^–1^	log *K*_*N*_ (298)/M^–1^
1	272	–26	–9	–16	2.9
2	318	–37	–17	–21	3.7
3	342	–42	–18	–24	4.3

The values of log *K*_*N*_ at 298 K in Table 2 are similar to the corresponding values from the titration data (Table 1), which suggests that the assumptions used in fitting the thermal denaturation data are reasonable. The experimental data in Fig. 6 clearly follow the theoretical sigmoidal curve corresponding to the melting transition. The free-bound transitions occur over narrower temperature ranges with increasing *N*, and the point of inflection occurs at higher temperatures. These visual observations are confirmed by the calculated parameters shown in Table 2, which show that the increased binding constants observed for higher values of *N* are associated with an increase in the enthalpy change on duplex formation and with an increase in the transition melting temperature. These features are characteristic of cooperative interactions between the H-bonding sites along the duplex.^10^

## Conclusions

We present a general strategy for the design of synthetic information molecules that could potentially encode and replicate chemical information in the same way as nucleic acids. A series of H-bond donor (phenol) and H-bond acceptor (phosphine oxide) oligomers (D_*N*_ and A_*N*_, *N* = 1–4) have been synthesised using reductive amination chemistry. The assembly of duplexes, A_*N*_·D_*N*_, was characterised using NMR titrations and thermal denaturation experiments in toluene. The stability of the duplex increases by one order of magnitude for every H-bonding group added to the chain. Similarly, the enthalpy change for duplex assembly and the melting temperature for duplex denaturation both increase with increasing chain length. Although the oligomers have a relatively flexible backbone, this lack of preorganisation does not significantly impede assembly of the duplex, and H-bond formation along the oligomers is cooperative. The effective molarity for intramolecular H-bond formation (EM = 14 mM) appears to be sufficient to propagate the formation of long duplexes using this approach. The product *K* EM, which is used to quantify chelate cooperativity is 5, which means that each H-bond is more than 80% populated in the assembled duplex. The modular design of these compounds should allow us to explore variations in the recognition and backbone linker modules to optimise the properties of the system for selective recognition of mixed sequence oligomers and template-directed synthesis.

## Supplementary Material

Supplementary information
