# Supplementary material for: Cooperative duplex formation by synthetic H-bonding oligomers
Source: Chem Sci. 2015 Oct 22;7(1):94–101. doi: 10.1039/c5sc03414k (PMC5950798; doi:10.1039/c5sc03414k)
Supplement: Supplementary file 1 [file SC-007-C5SC03414K-s001.pdf]

Electronic Supplementary Material (ESI) for Chemical Science.
This journal is © The Royal Society of Chemistry 2015

Supporting information

Cooperative Duplex Formation by Synthetic H-Bonding Oligomers

Alexander E. Stross, Giulia Iadevaia, Christopher A. Hunter*

Table of Contents

		Page
Section S1	NMR titrations	S2
Section S2	Variable temperature NMR	S9
Section S3	Synthesis	S11
Section S4	References	S38

*Department of Chemistry, University of Cambridge, Lensfield Road, Cambridge CB2 1EW

S1: NMR titrations

All binding constants were measured by NMR titrations. The host was dissolved in toluene-d_8 at a known concentration. The guest is dissolved in the host solution and made to a known concentration. A known volume of host is added to an NMR tube and the spectrum was recorded. Known volumes of guest in host solution were added to the NMR tube, and the spectra were recorded after each addition. The chemical shifts of the host spectra were monitored as a function of guest concentration and analysed using a purpose written software in Microsoft Excel. Errors were calculated as two times the standard deviation from the average value.

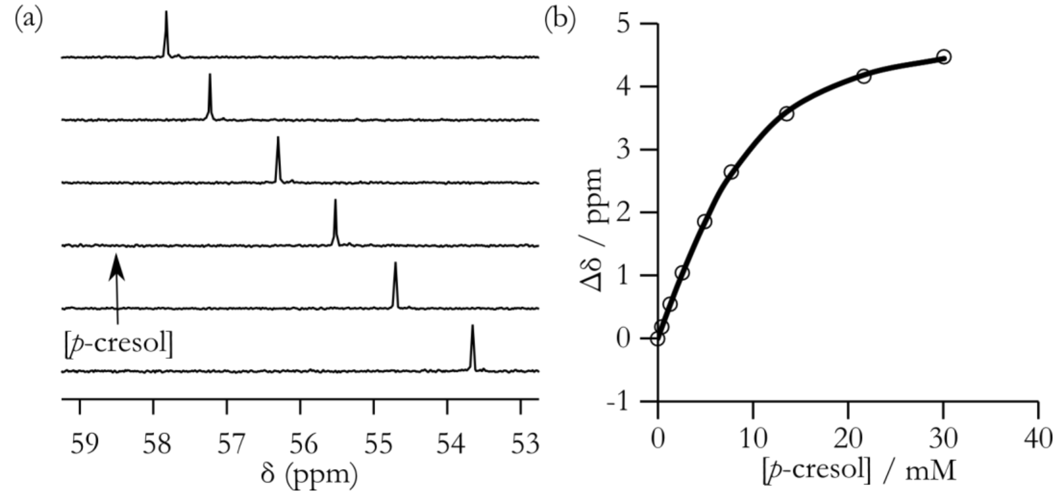

Figure S1. ^{1}H NMR data for titration of *p*-cresol into **5** (9.1 mM) at 298 K in toluene-d_8. (a) Example 162 MHz ^{31}P NMR spectra. (b) Plot of the change in chemical shift of the ^{31}P signal as a function of guest concentration (the line represents the best fit to a 1:1 binding isotherm).

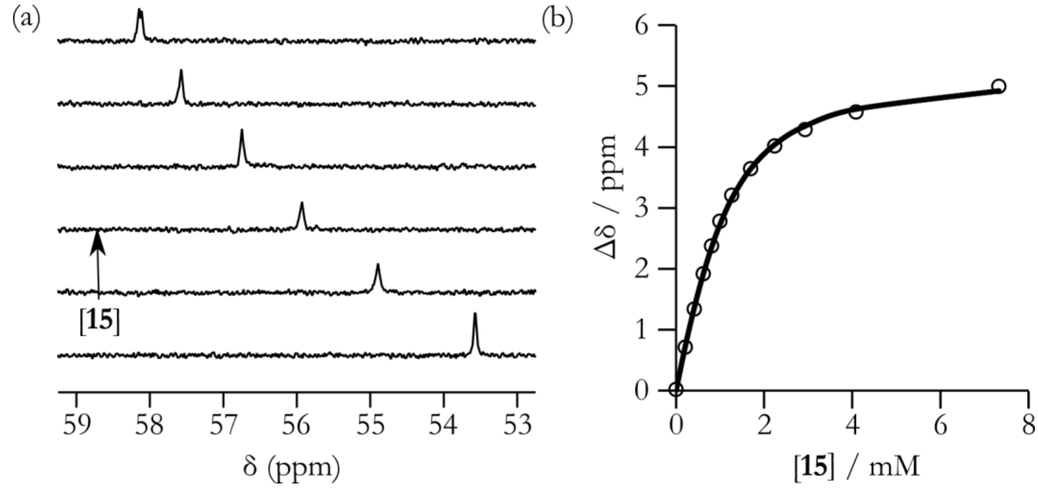

Figure S2. ^{31}P NMR data for titration of **15** into **14** (2.0 mM) at 298 K in toluene-d_8. (a) Example 162 MHz ^{31}P NMR spectra. (b) Plot of the change in chemical shift of the ^{31}P signal as a function of guest concentration (the line represents the best fit to a 1:1 binding isotherm).

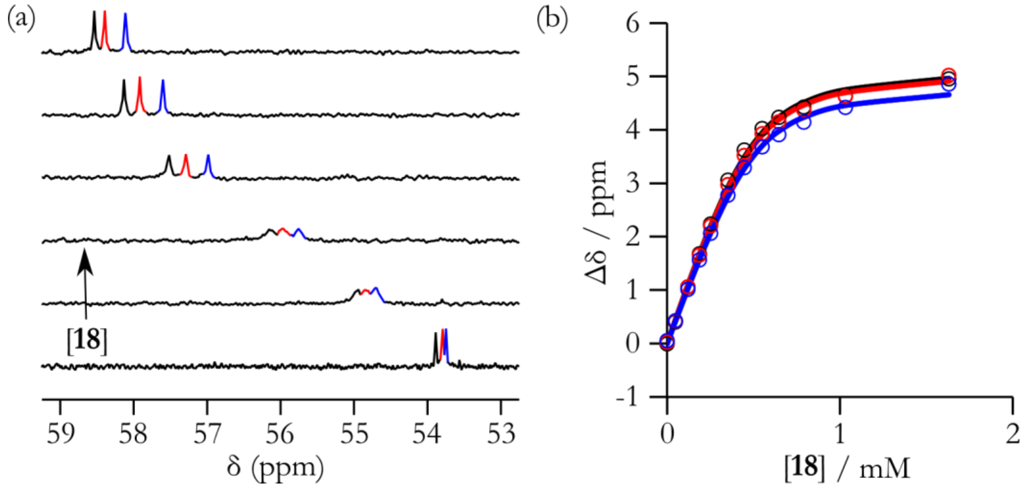

Figure S3. ^{31}P NMR data for titration of **18** into **17** (0.5 mM) at 298 K in toluene-d_8. (a) Example 162 MHz ^{31}P NMR spectra. (b) Plot of the change in chemical shift of the ^{31}P signals as a function of guest concentration (the line represents the best fit to a 1:1 binding isotherm).

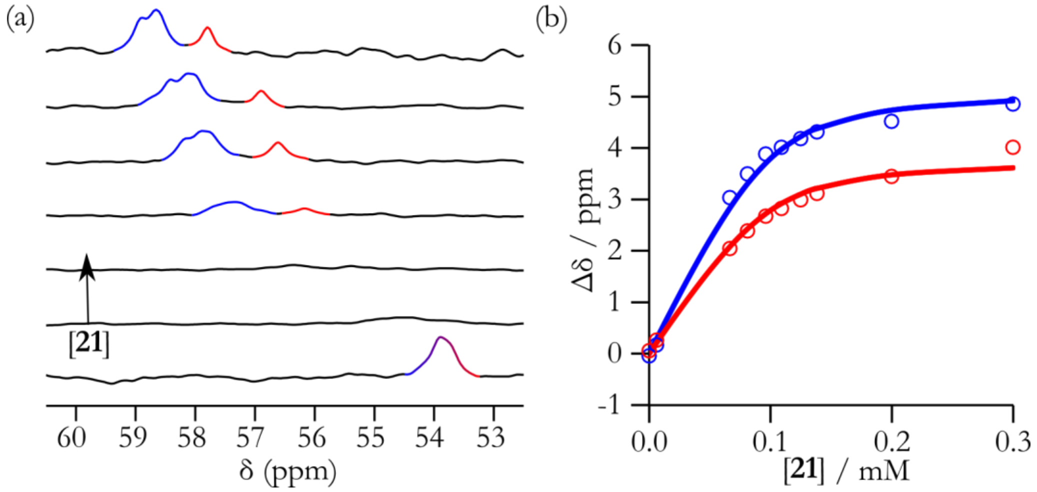

Figure S4. ^{31}P NMR data for titration of **21** into **20** (0.1 mM) at 298 K in toluene-d_8. (a) Example 162 MHz ^{31}P NMR spectra. (b) Plot of the change in chemical shift of the ^{31}P signals as a function of guest concentration (the line represents the best fit to a 1:1 binding isotherm). Due to the low host concentration and peak broadening throughout the titration a high line broadening (40 Hz) was used to monitor three of the four ^{31}P chemical shifts as one broad peak.

Table S1. ^{31}P NMR chemical shifts of free host (ppm) obtained by fitting titration data measured in toluene-d_8 at 298 K to a 1:1 binding isotherm.

A···D	[A···D<br>A···D]	[A···D<br>A···D<br>A···D]	[A···D<br>A···D<br>A···D<br>A···D]
**5**•*p*-cresol	**14•15**	**17•18**	**20•21**
53.7	53.6	53.9	53.9
	53.6	53.8	53.9
		53.7	53.9
			53.8

Table S2. Limiting complexation-induced ^{31}P NMR chemical shifts of fully bound host (ppm) obtained by fitting titration data measured in toluene-d_8 at 298 K to a 1:1 binding isotherm.

A···D	[A···D<br>A···D]	[A···D<br>A···D<br>A···D]	[A···D<br>A···D<br>A···D<br>A···D]
**5**•*p*-cresol	**14•15**	**17•18**	**20•21**
58.7	58.8	59.0	59.0
	58.8	58.7	59.0
		58.5	59.0
			57.6

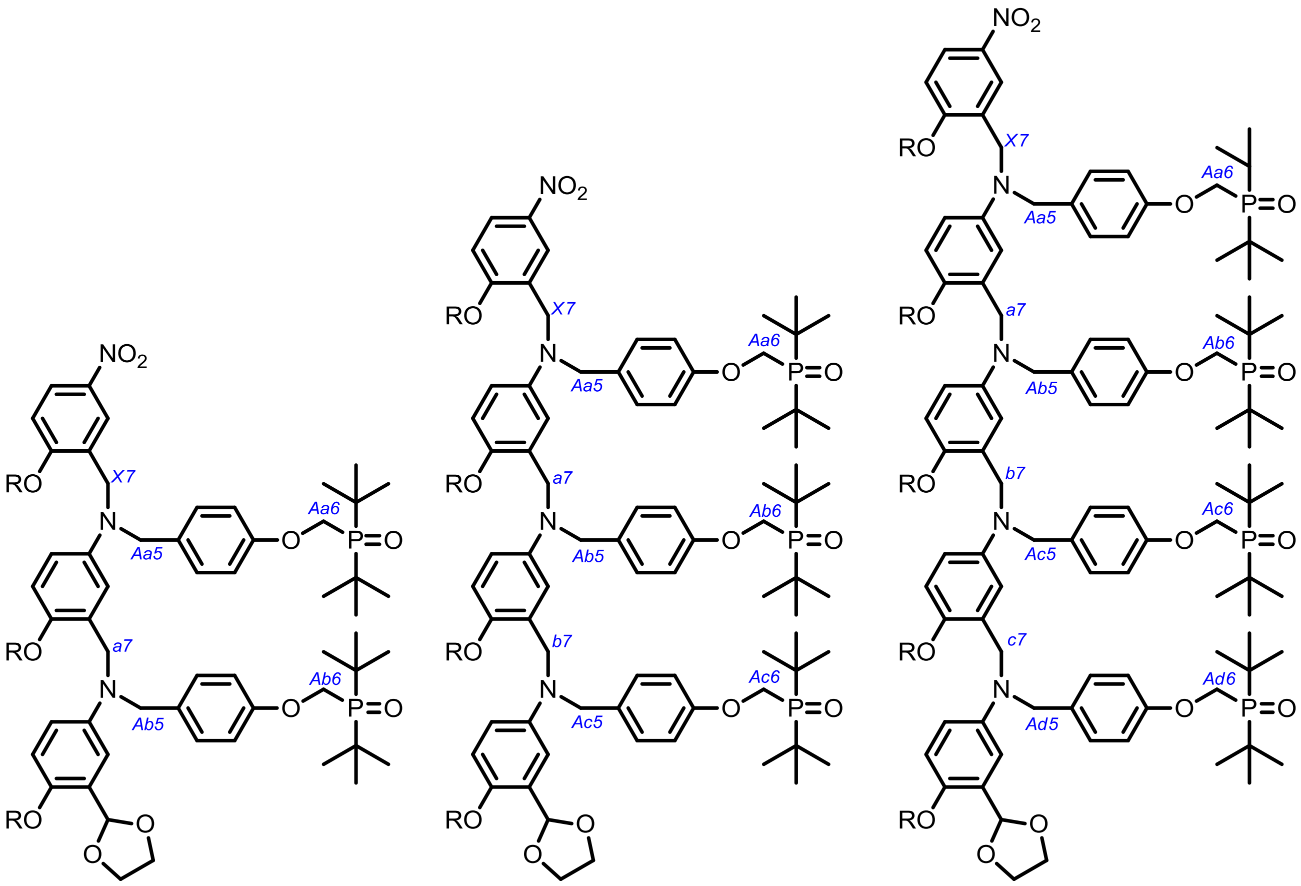

14 **17** **20**

Figure S5. Proton labelling scheme. R=2-ethylhexyl

Table S3. ^{1}H NMR chemical shifts of the free host (ppm) obtained by fitting titration data measured in toluene-d_8 at 298 K to a 1:1 binding isotherm.[a]

proton label	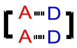 **14•15**	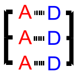 **17•18**	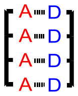 **20•21**
Aa6	4.2	4.2	4.3
Ab6	4.1	4.3	4.2
Ac6		4.2	4.4
Ad6			4.2
X7	4.5	4.6	4.5
a7	4.6	4.7	4.7
b7		4.7	4.7
c7			4.7
Aa5	4.4	4.4	4.3
Ab5	4.3	4.1	4.3
Ac5		4.1	4.1
Ad5			4.1

[a] refer to Figure S5 for proton labelling scheme

Table S4. Limiting complexation-induced ^{1}H NMR chemical shifts of the free host (ppm) obtained by fitting titration data measured in toluene-d_8 at 298 K to a 1:1 binding isotherm.[a]

proton label	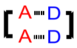 **14•15**	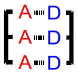 **17•18**	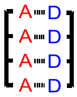 **20•21**
Aa6	4.3	4.4	4.5
Ab6	4.2	4.5	4.3
Ac6		4.4	4.6
Ad6			[b]
X7	4.5	4.6	4.5
a7	4.6	4.8	[b]
b7		4.8	[b]
c7			[b]
Aa5	4.3	4.3	[b]
Ab5	4.3	4.0	[b]
Ac5		4.1	4.0
Ad5			4.1

[a] refer to Figure S5 for numbering scheme; [b] signal could not be monitored due to signal overlap.

Table S5. Limiting complexation-induced changes in ^{1}H NMR chemical shift changes (ppm) obtained by fitting titration data measured in toluene-d_8 at 298 K to a 1:1 binding isotherm.[a]

proton label	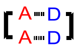 **14•15**	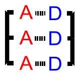 **17•18**	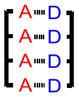 **20•21**
Aa6	0.1	0.2	0.2
Ab6	0.1	0.2	0.1
Ac6		0.2	0.2
Ad6			
X7	0.0	0.1	0.0
a7	0.0	0.1	[b]
b7		0.1	[b]
c7			[b]
Aa5	-0.1	0.0	[b]
Ab5	0.0	-0.1	[b]
Ac5		-0.1	-0.1
Ad5			0.0

[a] refer to Figure S5 for numbering scheme; [b] signal could not be monitored due to signal overlap.

Section S2: Variable temperature NMR

The temperature of the sample was changed using the internal thermostat of the NMR spectrometer, and the sample was allowed to equilibrate in the probe until the probe thermometer gave a stable temperature. The two chemically different ^{31}P signals in **14•15** gave a single overlapping peak that began to split into two signals at lower temperatures, so for high temperatures the chemical shift at the peak maximum was used, and at lower temperatures, the average of the chemical shifts of the two peaks was used (Figure S6 (b)). Due to significant broadening of the ^{31}P NMR peaks for **17•18** 40 Hz line broadening was applied to all spectra. At high temperatures where one broad peak was observed, the chemical shift was recorded at the highest point of the peak. At lower temperatures, two of the three separate signals were resolved, so the average of the chemical shifts of the peaks was used (Figure S6(c)). Even after applying the same processing (40 Hz line broadening) to the spectra of **30•27** the peaks were too broad at lower temperatures to reliably distinguish from the background noise. (Figure S6(d)). Figure S6(e) shows the ^{31}P NMR spectra of phosphine oxide 1-mer **5** only (1 mM) at various temperatures.

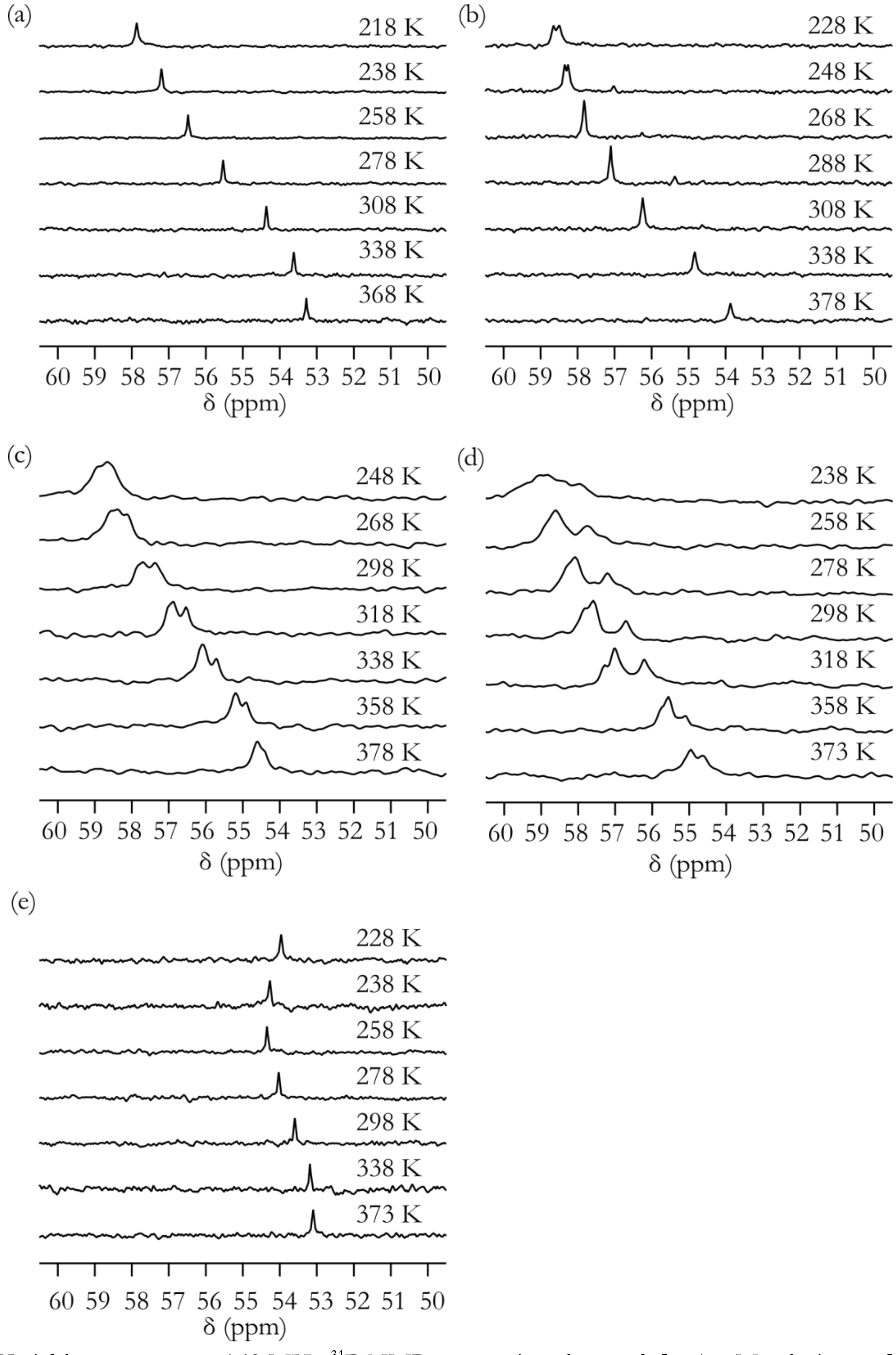

Figure S6. Variable temperature 162 MHz ^{31}P NMR spectra in toluene-d_8 for 1 mM solutions of 1:1 mixtures of (a) *p*-cresol•**5**; (b) **14•15**; (c) **17•18**; (d) **20•21;** and (e) only **5**. A high line broadening (40 Hz) was applied to the spectra in (c) and (d).

Section S3: Synthesis

Synthesis of **1**[1]

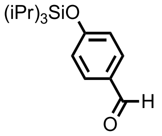

4-Hydroxybenzaldehyde (5.01 g, 40.9 mmol, 1 equiv.) was dissolved in DMF (50 mL) and TiPS-Cl (10.1 mL, 47.1 mmol, 1.2 equiv.) and imidazole (5.58 g, 81.9 mmol, 2 equiv.) were added with stirring. After 12 hours stirring, the reaction mixture was poured onto water (100 mL), before extraction into hexane (5 × 20 mL). The organic fractions were washed several times with water (5 × 100 mL) then brine (1 × 100 mL), dried ($MgSO_4$) and the solvent was removed with a rotary evaporator to yield an oil (12.8 g). The crude product was purified by flash chromatography on silica eluting with 10% EtOAc in hexane to yield a pale yellow oil (11.0 g, 96%).

^{1}H NMR (250 MHz, $CDCl_3$): δ_H = 9.89 (s, 1H), 7.79 (d, 2H, *J* = 9.0), 6.99 (d, 2H, *J* = 9.0), 1.20 – 1.38 (m, 3H), 1.07 – 1.16 (m, 18H);

^{13}C NMR (63 MHz, $CDCl_3$): δ_C = 190.8, 161.9, 131.9, 130.2, 120.3, 17.8, 12.7;

MS (ES+): m/z (%) = 279 (100) [M + H^+];

HRMS (ES+): calculated for $C_{16}H_{27}O_2Si$ 279.1780, found 279.1791;

FT-IR (thin film): ν_{max}/cm^{-1} 2945, 2893, 2867, 2730, 1698, 1596, 1575, 1508, 1463, 1275, 1211, 1155.

Synthesis of **2**[2]

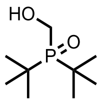

Di-*tert*-butylchlorophosphine (20.0 g, 111 mmol) was dissolved in 37% aqueous formaldehyde (210 mL) and concentrated aqueous HCl (200 mL) was added and heated to 100 °C for 12 hours with stirring. The reaction mixture was cooled to room temperature before being neutralised using NaOH. The aqueous solution was washed with EtOAc (3 × 100 mL). The organic fractions were washed with brine (1 × 100 mL), dried ($MgSO_4$) and the solvent removed using a rotary evaporator. The crude product was recrystallized in boiling hexane to yield a white solid (11.1 g, 52%).

^{1}H NMR (250 MHz, $CDCl_3$): δ_H = 4.05 (s, 2H), 1.28 (d, 18H, *J* = 13);

^{13}C NMR (63 MHz, $CDCl_3$): δ_C = 54.8 (d, *J* = 62), 34.8 (d, *J* = 55), 26.4;

^{31}P NMR (101 MHz, $CDCl_3$): δ_P = 154.0;

MS (ES+): m/z (%) = 193 (100) [M + H^+];

HRMS (ES+): calculated for $C_9H_{22}O_2P$ 193.1357, found 193.1367;

FT-IR (thin film): ν_{max}/cm^{-1} 3142, 2957, 1474, 1129, 1120, 1064.

Synthesis of **3**[2]

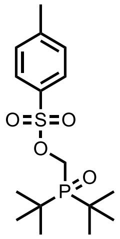

2 (9.2 g, 48 mmol, 1 equiv.) was dissolved in THF 130 mL and NEt_3 (10 mL) at 0 °C with stirring. 4-toluenesulfonyl chloride (11.4 g, 60 mmol, 1.3 equiv.) in THF (30 mL) was added slowly over 20 minutes. After 18 hours the mixture was poured onto water (100 mL) and extracted into EtOAc (3 × 20 mL). The organic extracts were washed with water (1 × 20 mL) and brine (1 × 2 mL) before drying ($MgSO_4$). The solvent was removed using a rotary evaporator and the crude product purified by flash chromatography on silica eluting with a gradient from 0% to 3% MeOH in DCM to yield a colourless oil, which after several days crystallised into a white amorphous solid (14.4 g, 87%).

^{1}H NMR (250 MHz, $CDCl_3$): δ_H = 7.81 (d, 2H, *J* = 8.0), 7.39 (d, 2H, *J* = 8.0), 4.27 (d, 2H, *J* = 7.0), 2.47 (s, 3H), 1.29 (d, 18H, *J* = 13.8);

^{13}C NMR (63 MHz, $CDCl_3$): δ_C = 145.7, 131.1, 130.0, 128.2, 61.4 (d, *J* = 60.5), 35.6 (d, *J* = 58.5), 26.2, 21.6;

^{31}P NMR (101 MHz, $CDCl_3$): δ_P = 56.0;

MS (ES+): m/z (%) = 347 (100) [M + H^+];

HRMS (ES+): calculated for $C_{16}H_{28}O_4PS$ 347.1446, found 347.1456;

FT-IR (thin film): ν_{max}/cm^{-1} 3431, 2968, 2874, 1597, 1478, 1369, 1190, 1176, 1146, 1095, 996.

Synthesis of **4**

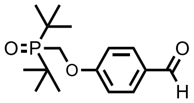

4-hydroxybenzaldehye (0.55 g, 4.5 mmol, 1 equiv.) was dissolved in DMF (20 mL) and **3** (1.70 g, 4.9 mmol, 1.1 equiv.) and Cs_2CO_3 (2.18 g, 6.69 mmol, 1.5 equiv.) were added and the mixture heated at 80 °C for 3 days with stirring. The reaction was then poured onto water (100 mL) and extracted into EtOAc (5 × 20 mL), before being washed with water (5 × 40 mL) and brine (1 × 40 mL) and then dried ($MgSO_4$). The solvent was removed using a rotary evaporator before the crude product was purified using flash chromatography on silica eluting with a gradient from 0% to 10% MeOH in diethyl ether to yield an amorphous white solid (0.88 g, 66%).

^{1}H NMR (250 MHz, $CDCl_3$): δ_H = 9.93 (s, 1H), 7.89 (d, 2H, *J* = 9.0), 7.07 (d, 2H, *J* = 9.0), 4.48 (d, 2H, *J* = 7.0), 1.40 (d, 18H, *J* = 14.0);

^{13}C NMR (63 MHz, $CDCl_3$): δ_C = 190.2, 162.8 (d, *J* = 9.6), 131.8, 130.7, 114.33, 62.6 (d, *J* = 68.1), 35.2 (d, *J* = 57.6), 26.2;

^{31}P NMR (101 MHz, $CDCl_3$): δ_P = 56.5;

MS (ES+): m/z (%) = 297 (100) [M + H^+], 338 (15) [M + CH_3CN], 360 (10);

HRMS (ES+): calculated for $C_{16}H_{26}O_3P$ 297.1620, found 297.1619;

FT-IR (thin film): ν_{max}/cm^{-1} 2967, 2906, 2871, 1694, 1603, 1582, 1509, 1306, 1242, 1163, 1133, 1041.

Synthesis of **5**

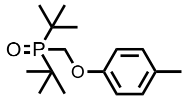

p-Cresol (0.21 g, 1.9 mmol, 1 equiv.) was dissolved in DMF (10 mL) and **3** (0.73 g, 2.1 mmol, 1.1 equiv.) and Cs_2CO_3 (0.93 g, 2.86 mmol, 1.5 equiv.) were added and stirred at 80 °C for 12 h. The reaction mixture was poured onto water (100 ml) and extracted into EtOAc (5 × 20 mL), before being washed with water (5 × 20 mL), brine (1 × 20 mL) and dried ($MgSO_4$). The solvent was removed using a rotary evaporator and the crude product was purified via flash chromatography on silica eluting with a gradient from 0% to 10% MeOH in EtOAc to yield a white amorphous solid (0.21 g, 39%).

^{1}H NMR (250 MHz, $CDCl_3$): δ_H = 7.12 (d, 2H, *J* = 8.5), 6.83 (d, 2H, *J* = 8.5), 4.37 (d, 2H, *J* = 8.0), 2.30 (s, 3H), 1.39 (d, 18H, *J* = 13.5);

^{13}C NMR (63 MHz, $CDCl_3$): δ_C = 156.3 (d, *J* = 10.5), 131.1, 130.1, 113.1, 62.6 (d, *J* = 70.5), 35.4 (d, *J* = 56.5), 26.4, 20.4;

^{31}P NMR (101 MHz, $CDCl_3$): δ_P = 56.8;

MS (ES+): m/z (%) = 283 (100) [M + H^+], 346 (50), 383 (15);

HRMS (ES+): calculated for $C_{16}H_{28}O_2P$ 283.1827, found 283.1840;

FT-IR (thin film): ν_{max}/cm^{-1} 3431, 2955, 2874, 1597, 1478, 1368, 1190, 1176, 1145, 996.

Synthesis of **6**

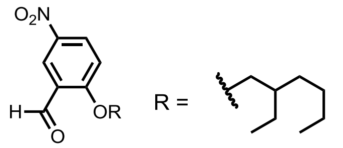

2-Hydroxy-5-nitrobenzaldehyde (15 g, 90 mmol, 1 equiv.), K_2CO_3 (18.6 g, 135 mmol, 1.5 equiv.) and 2-ethylhexyl bromide (23.9 mL, 135 mmol, 1.5 equiv.) were dissolved in DMF (100 mL) and heated to 70 °C for 4 days with stirring. The mixture was then poured onto water (100 mL) and extracted into ethyl acetate (3 × 100 mL), before being washed with water (5 × 200 mL) and finally brine (1 × 100 mL). The organic fractions were dried ($MgSO_4$) and the solvent removed using a rotary evaporator to give the crude oil (38.8 g), which was purified by flash chromatography on silica eluting with 10% ethyl acetate in hexane to give product yellow oil (23.0 g, 91%).

^{1}H NMR (250 MHz, $CDCl_3$): δ_H = 10.48 (s, 1H), 8.70 (d, 1H, *J* = 3.0), 8.42 (dd, 1H, *J* = 9.0, 3.0), 7.12 (d, 1H, *J* = 9.0), 4.11 (d, 2H, *J* = 6.0), 1.86 (s, 1H), 1.23 – 1.64 (m, 8H), 0.84 – 1.03 (m, 6H);

^{13}C NMR (63 MHz, $CDCl_3$): δ_C = 187.4, 165.4, 141.4, 124.7, 124.4, 112.9, 72.3, 39.3, 30.4, 29.0, 23.9, 22.9, 13.9, 11.0;

MS (ES+): m/z (%) = 280 (100) [M + H^+], 291 (20).

HRMS (ES+): calculated for $C_{15}H_{22}NO_4$ 280.1549, found 280.1541;

FT-IR (thin film): ν_{max}/cm^{-1} 2960, 2931, 2873, 1692, 1608, 1590, 1522, 1488, 1461, 1429, 1176, 1142, 1077, 1005.

Synthesis of **7**

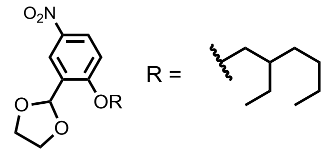

6 (14.1 g, 50.6 mmol, 1 equiv.) was dissolved in toluene (75 mL) and *p*-toluenesulfonic acid (0.19 g, 1.01 mmol, 0.02 equiv.) and ethylene glycol (9.43, 151 mmol, 3 equiv.) were added and the solution heated to reflux with stirring. The toluene/water condensate was removed using Dean-Stark apparatus until the condensate ran clear. The reaction was cooled to room temperature and poured onto water (100 mL) before being extracted into EtOAc (3 × 50 mL). The organic fractions were collected and washed with brine (1 × 100 mL), dried ($MgSO_4$), and concentrated on a rotary evaporator. The crude product required no further purification and was isolated as a golden viscous oil (15.9 g, 96% yield).

^{1}H NMR (250 MHz, $CDCl_3$): δ_H = 8.43 (d, 1H, *J* = 3.0), 8.24 (dd, 1H, *J* = 9.0, 3.0), 6.96 (d, 1H, *J* = 9.0), 6.14 (s, 1H), 4.03 – 4.22 (m, 4H), 4.01 (d, 2H, *J* = 5.5), 1.73 – 1.89 (m, 1H), 1.24 – 1.61 (m, 8H), 0.87 – 1.00 (m, 6H);

^{13}C NMR (63 MHz, $CDCl_3$): δ_C = 162.3, 141.1, 127.5, 126.4, 123.3, 111.2, 98.3, 71.8, 65.4, 39.2, 30.4, 29.0, 14.0, 11.0;

MS (ES+): m/z (%) = 324 (100) [M + H^+].

HRMS (ES+): calculated for $C_{17}H_{26}NO_6$ 324.1811, found 324.1820.

FT-IR (thin film): ν_{max}/cm^{-1} 2959, 2931, 2875, 1613, 1596, 1518, 1494, 1463, 1340, 1270, 1105, 1067.

Synthesis of **8**

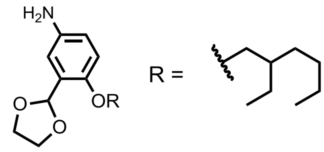

7 (30.56 g, 94.51 mmol, 1 equiv., 0.2 M) was dissolved in in degassed EtOAc (475 mL) and Pd/C (2.01 g, 18.9 mmol) was added with rigorous stirring. The reaction was stirred in an atmosphere of H_2 overnight. The reaction is then filtered through a plug of Celite and the solvent was removed using a rotary evaporator to yield a dark red oil. The crude product was purified by flash chromatography on silica eluting with a gradient from 30% to 40% of EtOAc/hexane. The product was isolated as a yellow oil (23.4 g, 84%).

^{1}H NMR (250 MHz, CDCl$_3$): δ_H = 6.94 (d, 1H, *J* = 3.0), 6.76 (d, 1H, *J* = 8.5), 6.69 (dd, 1H, *J* = 8.50, 3.0), 6.12 (s, 1H), 3.98 – 4.18 (m, 4H), 3.82 (d, 2H, *J* = 5.5), 1.64 – 1.82 (m, 1H), 1.22 – 1.59 (m, 8H), 0.85 – 0.97 (m, 6H);

^{13}C NMR (63 MHz, CDCl$_3$): δ_C = 150.9, 139.5, 127.0, 117.0, 114.2, 113.8, 99.2, 72.0, 65.2, 39.5, 30.5, 29.1, 23.9, 23.0, 14.0, 11.1.

MS (ES+): m/z (%) = 294 (100) [M + H$^+$];

HRMS (ES+): calculated for $C_{17}H_{28}NO_3$ 294.2069, found 294.2055;

FT-IR (thin film): ν_{max}/cm^{-1} 3360, 2957, 2927, 2873, 1627, 1501, 1455, 1383, 1264, 1221, 1172, 1067.

Synthesis of **9**

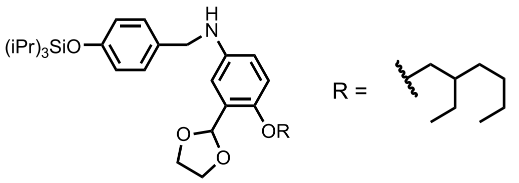

8 (4.92 g, 16.8 mmol, 1 equiv.) and **4** (5.14 g, 18.5 mmol, 1.1 equiv.) were dissolved in toluene (30 mL) with stirring and heated under Dean-Stark apparatus until the condensate ran clear. The solvent was removed using a rotary evaporator and the crude mixture was dissolved in MeOH (60 mL) and diethyl ether (30 mL) and $NaBH_4$ (1.90 g, 50.3 mmol, 3 equiv.) was added slowly at 0 °C with stirring. This mixture was stirred for 2 hours before the solution was neutralised using concentrated aqueous HCl. This solution was washed with EtOAc (5 × 30 mL) and all the organic extracts were combined and washed with brine (1 × 20 mL) and then dried ($MgSO_4$). The crude oil was purified via flash chromatography on silica eluting with a gradient from 0% to 10% EtOAc in hexane to yield a yellow oil (8.07 g, 87%).

^{1}H NMR (250 MHz, $CDCl_3$): δ_H = 7.21 (d, 2H, *J* = 8.5), 6.89 (d, 1H, *J* = 3.0), 6.85 (d, 2H, *J* = 9.0), 6.78 (d, 1H, *J* = 9.0), 6.60 (dd, 1H, *J* = 9.0, 3.0), 6.14 (s, 1H), 4.20 (s, 2H), 3.97 – 4.14 (m, 4H), 3.82 (d, 2H, *J* = 6.0), 1.74 (s, 1H), 1.20 – 1.56 (m, 11H), 1.11 (d, 18H, *J* = 7.0), 0.87 – 0.98 (m, 6H);

^{13}C NMR (63 MHz, $CDCl_3$): δ_C = 155.2, 150.2, 142.2, 131.9, 128.8, 127.1, 119.9, 114.4, 113.9, 112.1, 99.4, 72.0, 65.1, 48.9, 39.5, 30.5, 29.1, 23.9, 23.1, 17.9, 14.0, 12.7, 11.1;

MS (ES+): m/z (%) = 556 (100) [M + H^+];

HRMS (ES+): calculated for $C_{33}H_{54}NO_4Si$ 556.3822, found 556.3800;

FT-IR (thin film): ν_{max}/cm^{-1} 2928, 2866, 1608, 1506, 1465, 1263, 1227, 1169, 1069, 913.

Synthesis of **10**

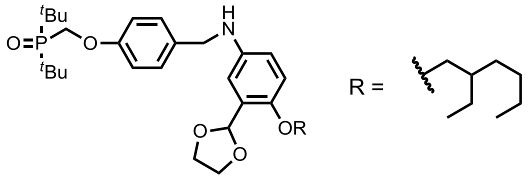

4 (0.79 g, 2.7 mmol, 1 equiv.) and **8** (1.02 g, 3.5 mmol, 1.3 equiv.) were dissolved in toluene (50 mL) with stirring and heated under Dean-Stark apparatus until the condensate ran clear. The solvent was removed with a rotary evaporator and the crude mixture was dissolved in MeOH (20 mL) and $NaBH_4$ (0.61 g, 16.0 mmol, 6 equiv.) was added slowly at 0 °C with stirring. After 2 hours the solution was neutralised using concentrated aqueous HCl. This solution was washed with EtOAc (5 × 20 mL) and all the organic extracts were combined and washed with brine (1 × 20 mL) and then dried ($MgSO_4$) and the solvent removed with a rotary evaporator. The crude oil was purified via flash chromatography on silica eluting with a gradient from 0% to 10% MeOH in EtOAc to yield a yellow oil (0.87 g, 57%).

^{1}H NMR (250 MHz, $CDCl_3$): δ_H = 7.25 (d, 2H, *J* = 8.5), 6.82 – 6.85 (m, 3H), 6.70 (d, 1H, *J* = 9.0), 6.51 (dd, 1H, *J* = 9.0, 3.0), 6.07 (s, 1H), 4.32 (d, 2H, *J* = 7.5), 4.16 (s, 2H), 3.90 – 4.05 (m, 4H), 3.75 (d, 2H, *J* = 6.0), 1.63 – 1.71 (m, 1H), 1.20 – 1.50 (m, 26H), 0.83 – 0.91 (m, 6H);

^{13}C NMR (63 MHz, $CDCl_3$): δ_C = 157.2 (d, *J* = 10.6), 149.7, 141.8, 132.9, 128.6, 126.7, 113.8, 113.8, 113.5, 111.8, 99.0, 71.6, 64.8, 62.3 (d, *J* = 71.0), 48.1, 39.2, 35.1 (d, *J* = 57.6), 30.2, 28.7, 26.1, 22.7, 13.8, 10.8;

^{31}P NMR (101 MHz, $CDCl_3$): δ_P = 56.6;

MS (ES+): m/z (%) = 574 (100) [M + H^+], 1148 (15);

HRMS (ES+): calculated for $C_{33}H_{53}NO_5P$ 574.3661, found 574.3642;

FT-IR (thin film): ν_{max}/cm^{-1} 2956, 2927, 2871, 1691, 1601, 1506, 1468, 1392, 1226, 1136, 1069, 1040.

Synthesis of **11**

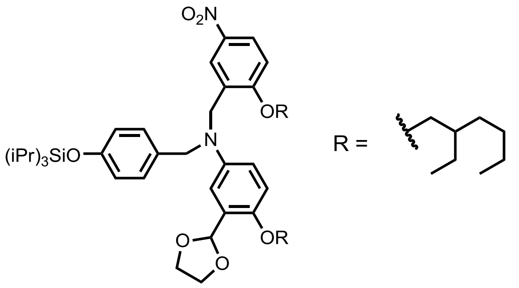

9 (2.11 g, 3.8 mmol, 1 equiv.) and **6** (1.77 g, 6.3 mmol, 1.2 equiv.) were dissolved in DCE (20 mL) and $NaBH(OAc)_3$ (3.13 g, 2.8 mmol, 1.2 equiv.) was added with stirring. After 12 hours the reaction was quenched with saturated aqueous $NaHCO_3$ solution, and extracted into $CHCl_3$ (4 × 20 mL). All the organic fractions were washed with water (1 × 20 mL), brine (1 × 10 mL) and dried ($MgSO_4$) before the solvent removed with a rotary evaporator. The crude product was purified using flash chromatography on silica eluting with a gradient from 0% to 10% of EtOAc in hexane to yield a golden yellow oil (2.48 g, 79%).

^{1}H NMR (400 MHz, $CDCl_3$): δ_H = 8.08 – 8.15 (m, 2H), 7.12 (d, 2H, *J* = 8.5), 6.97 (d, 1H, *J* = 3.0), 6.90 (d, 1H, *J* = 9.5), 6.81 (d, 2H, *J* = 8.5), 6.74 (d, 1H, *J* = 9.0), 6.60 (dd, 1H, *J* = 9.0, 3.0), 6.14 (s, 1H), 4.55 (s, 2H), 4.49 (s, 2H), 3.97 (d, 2H, *J* = 6.5), 3.94 (s, 4H), 3.80 (d, 2H, *J* = 6.0), 1.67 – 1.84 (m, 2H), 1.17 – 1.55 (m, 19H), 1.09 (d, 18H, *J* = 7.0); 0.86 – 0.97 (m, 12H);

^{13}C NMR (101 MHz, $CDCl_3$): δ_C = 161.8, 155.0, 149.9, 143.0, 141.40, 130.8, 128.80, 128.20, 126.90, 124.3, 123.5, 119.9, 114.8, 113.5, 112.3, 110.2, 99.6, 71.6, 71.3, 65.0, 55.1, 49.8, 39.4, 39.2, 30.5, 29.0, 23.9, 23.1, 22.9, 17.9, 14.1, 14.0, 12.6, 11.1;

MS (ES+): m/z (%) = 820 (100) [M + H^+];

HRMS (ES+): calculated for $C_{48}H_{75}N_2O_7Si$ 819.5344, found 819.5344;

FT-IR (thin film): ν_{max}/cm^{-1} 2921, 2852, 1660, 1633, 1610, 1595, 1508, 1465, 1378, 1340, 1264, 1076.

Synthesis of **11'**

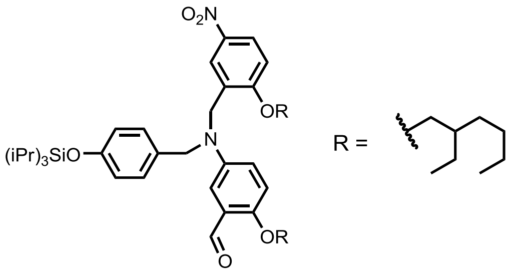

11 (1.39 g, 1.7 mmol, 1 equiv.) was dissolved in $CHCl_3$ (10 mL) and concentrated aqueous acid (10 mL) was added with stirring. After 18 hours the mixture was neutralised using aqueous $NaHCO_3$ and the organic portion separated from the aqueous part. The aqueous layer was washed with $CHCl_3$ (4 × 10 mL) before all organic fractions were washed with brine (1 × 10 mL) dried ($MgSO_4$) and the solvent removed with a rotary evaporator to yield a yellow oil (1.30 g, 99%) requiring no further purification.

^{1}H NMR (400 MHz, $CDCl_3$): δ_H = 10.45 (s, 1H), 8.14 (dd, 1H. *J* = 9.0, 3.0), 8.01 (d, 1H, *J* = 2.5), 7.17 (d, 1H, *J* = 3.0), 7.10 (d, 2H, *J* = 8.5), 6.89 – 6.94 (m, 2H), 6.80 – 6.87 (m, 3H), 4.57 (s, 2H), 4.54 (s, 2H), 3.99 (d, 2H, *J* = 5.5), 3.89 (d, 2H, *J* = 5.5), 1.69 – 1.84 (m, 2H), 1.17 – 1.59 (m, 19H), 1.09 (d, 18H, *J* = 7.0), 0.86 – 0.98 (m, 12H);

^{13}C NMR (63 MHz, $CDCl_3$): δ_C = 190.0, 161.9, 155.1, 154.6, 142.9, 141.4, 130.2, 128.0, 127.9, 125.3, 124.5, 123.0, 121.2, 120.1, 114.2, 110.9, 110.4, 71.5, 71.3, 54.7, 49.6, 39.5, 39.2, 30.6, 30.5, 29.1, 29.0, 24.0, 23.9, 23.0, 22.9, 17.9, 14.0, 12.6, 11.2, 11.1;

MS (ES+): m/z (%) = 263 (40), 775 (100) [M + H^+];

HRMS (ES+): calculated for $C_{46}H_{71}N_2O_6Si$ 775.5081, found 775.5060;

FT-IR (thin film): ν_{max}/cm^{-1} 2956, 2925, 2858, 1684, 1610, 1591, 1508, 1463, 1341, 1266.

Synthesis of **12**

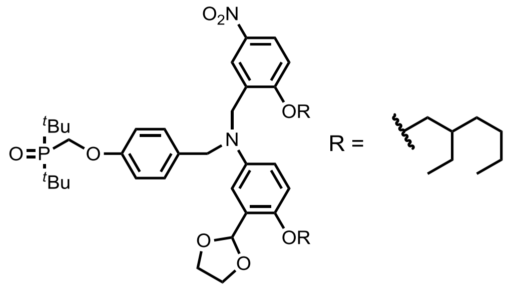

10 (0.160 g, 0.28 mmol, 1 equiv.) and **6** (0.156 g, 0.56 mmol, 2 equiv.) were dissolved in DCE (1 mL) and $NaBH(OAc)_3$ (0.165 g, 0.78 mmol, 2.8 equiv.) was added with stirring. After 18 hours the reaction was quenched with saturated aqueous $NaHCO_3$ solution, and extracted into $CHCl_3$ (4 × 10 mL). All the organic fractions were washed with water (1 × 10 mL), brine (1 × 10 mL) and dried ($MgSO_4$) before the solvent removed using a rotary evaporator. The crude product was purified using flash chromatography on silica eluting from 0% to 3% of MeOH in EtOAc to yield a pale yellow oil (0.22 g, 94%).

^{1}H NMR (500 MHz, $CDCl_3$): δ_H = 8.12 (dd, 1H, *J* = 9.0, 3.0), 8.10 (d, 1H, *J* = 3.0), 7.22 (d, 2H, *J* = 9.0), 6.97 (d, 1H, *J* = 3.0), 6.90 (d, 1H, *J* = 9.0), 6.86 (d, 2H, *J* = 9.0), 6.74 (d, 1H, *J* = 9.0), 6.61 (dd, 1H, *J* = 9.0, 3.0), 6.11 (s, 1H), 4.56 (s, 2H), 4.50 (s, 2H), (d, 2H, *J* = 7.5), 3.97 (d, 2H, *J* = 6.0), 3.93 – 3.99 (m, 4H), 3.79 (d, 1H, *J* = 6.0), 1.74 – 1.82 (m, 1H), 1.67 – 1.74 (m, 1H), 1.23 – 1.54 (m, 34H), 0.85 – 0.96 (m, 12H);

^{13}C NMR (126 MHz, $CDCl_3$): δ_C = 161.8, 157.4, (d, *J* = 10.6), 150.1, 142.7, 141.4, 132.0, 128.6, 128.5, 126.9, 124.3, 123.5, 115.1, 114.1, 113.5, 112.6, 110.3, 99.6, 71.6, 71.4, 65.0, 62.6 (d, *J* = 71.0), 55.0, 50.2, 39.5, 39.2, 35.4 (d, *J* = 57.6), 30.5, 29.0, 26.4, 23.9, 23.0, 22.9, 14.0, 11.1;

^{31}P NMR (101 MHz, $CDCl_3$): δ_P = 56.8;

MS (ES+): m/z (%) = 419 (10) [M + 2H$^+$], 838 (100) [M + H$^+$], 860 (45) [M + Na$^+$], 883 (15), 938 (30);

HRMS (ES+): calculated for $C_{48}H_{74}N_2O_8P$ 837.5183, found 837.5213;

FT-IR (thin film): ν_{max}/cm^{-1} 2957, 2922, 2853, 1738, 1610, 1592, 1508, 1466, 1340, 1265, 1231, 1139, 1078.

Synthesis of **12'**

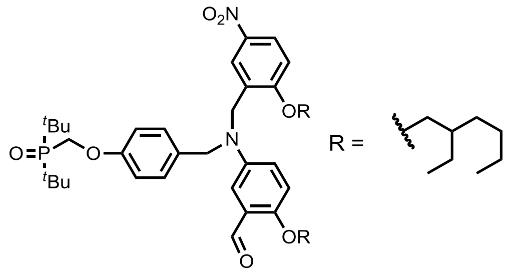

12 (0.22 g, 0.26 mmol, 1 equiv.) was dissolved in $CHCl_3$ (10 mL) and concentrated aqueous acid (10 mL) was added with stirring. After 18 hours the mixture was neutralised using aqueous $NaHCO_3$ and the organic portion separated from the aqueous part. The aqueous layer was washed with $CHCl_3$ (3 × 10 mL) before all organic fractions were washed with brine (1 × 10 mL) and dried ($MgSO_4$) and the solvent removed with a rotary evaporator to yield a yellow oil (0.20 g, 94%) requiring no further purification.

^{1}H NMR (400 MHz, $CDCl_3$): δ_H = 10.43 (s, 1H), 8.12 (dd, 1H, *J* = 9.0, 3.0), 7.97 (d, 1H, *J* = 3.0), 7.19 (d, 2H, *J* = 8.5), 7.14 (d, 1H, *J* = 3.0), 6.82 – 6.95 (m, 5H), 4.59 (s, 2H), 4.55 (s, 2H), 4.35 (d, 2H, *J* = 7.5), 3.99 (d, 2H, *J* = 5.5, 3.87 (d, 2H, *J* = 5.5), 1.66 – 1.83 (m, 2H), 1.21 – 1.56 (m, 34H), 0.83 – 0.96 (m, 12H);

^{13}C NMR (101 MHz, $CDCl_3$): δ_C = 189.9, 161.8, 157.4 (d, *J* = 11.6), 154.6, 142.5, 141.2, 131.1, 128.1, 127.6, 125.2, 124.5, 122.9, 121. 0, 114.2, 114.1, 110.8, 110.4, 71.4, 71.1, 62.5 (d, *J* = 70.9), 54.4, 49.8, 39.4, 39.1, 35. 3 (d, *J* = 57.0), 30.5, 30.4, 29.0, 28.9, 26.4, 23.9, 23.8, 22.9, 22.8, 14.0, 13.9, 11.1, 11.0;

^{31}P NMR (162 MHz, $CDCl_3$): δ_P = 56.6;

MS (ES+): m/z (%) = 397 (5) [M + 2H$^+$], 794 (100) [M + H$^+$], 816 (65) [M + Na$^+$], 839 (25), 864 (50);

HRMS (ES+): calculated for $C_{46}H_{70}N_2O_7P$ 793.4921, found 793.4921;

FT-IR (thin film): ν_{max}/cm^{-1} 2957, 2927, 2871, 1610, 1591, 1507, 1465, 1338, 1265, 1227, 1137, 1047, 1017.

Synthesis of **13**

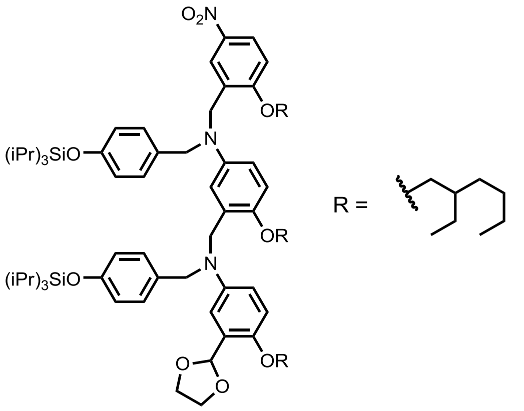

11' (2.02 g, 2.61 mmol, 1 equiv.) and **9** (2.90 g, 5.22 mmol, 2 equiv.) were dissolved in DCE (9 mL) and $NaBH(OAc)_3$ (1.55 g, 7.31 mmol, 2.8 equiv.) was added with stirring. After 1 day the reaction was quenched with saturated aqueous $NaHCO_3$ solution, and extracted into $CHCl_3$ (4 × 10 mL). All the organic fractions were washed with water (1 × 10 mL), brine (1 × 10 mL) and dried ($MgSO_4$) before the solvent was removed using a rotary evaporator. The crude product was purified using flash chromatography on silica eluting with a gradient from 0% to 10% of a 1:1 CHCl3/diethyl ether mix in hexane) to yield a golden yellow oil (1.86 g, 54%).

^{1}H NMR (400 MHz, $CDCl_3$): δ_H = 7.99 (dd, 1H, *J* = 9.0, 3.0), 7.92 (d, 1H, *J* = 3.0), 7.07 (d, 2H, *J* = 8.5), 6.99 (d, 2H, *J* = 8.5), 6.81 (d, 2H, *J* = 8.5), 6.76 (d, 2H, *J* = 8.5), 6.72 (d, 1H, *J* = 5.5), 6.67 – 6.70 (m, 2H), 6.49 – 6.54 (m, 2H), 6.37 (d, 1H, *J* = 3.0), 6.30 (dd, 1H, *J* = 9.0, 3.0), 6.10 (s, 1H), 4.49 (s, 2H), 4.39 – 4.45 (m, 4H), 4.18 (s, 2H), 3.85 – 3.93 (m, 6H), 3.73 – 3.80 (m, 4H), 1.68 – 1.79 (m, 2H), 1.60 – 1.68 (m, 1H), 1.16 – 1.54 (m, 27H), 1.08 (dd, *J* = 7.0, 2.0, 31H), 0.81 – 1.00 (m, 16H);

^{13}C NMR (101 MHz, $CDCl_3$): δ_C = 161.4, 154.9, 154.6, 149.1, 148.9, 143.1, 142.5, 141.2, 131.5, 131.3, 128.4, 128.1, 127.6, 127.2, 126.4, 124.2, 122.9, 119.9, 119.7, 113.4, 113.3, 112.9, 112.2, 111.3, 110.8, 110.4, 99.7, 71.7, 71.3, 70.7, 64.9, 55.6, 53.9, 50.1, 49.6, 39.6, 39.5, 39.2, 30.7, 30.5, 30.6, 29.1, 29.0, 24.0, 23.9, 23.1, 23.0, 17.9, 14.1, 14.0, 12.7, 11.2;

MS (ES+): m/z (%) = 1315 (80) [M + H^+], 1337 (100) [M + Na^+];

HRMS (ES+): calculated for $C_{79}H_{124}N_3O_9Si_2$ 1314.8876, found 1314.8842;

FT-IR (thin film): ν_{max}/cm^{-1} 2959, 2928, 2867, 1608, 1593, 1507, 1464, 1383, 1339, 1263, 1226, 1165, 1075, 1014.

Synthesis of **13'**

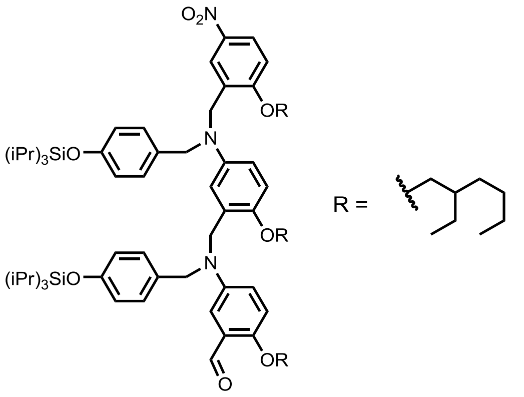

13 (0.68 g, 0.52 mmol, 1 equiv.) was dissolved in $CHCl_3$ (10 mL) and concentrated aqueous acid (10 mL) was added with stirring. After 18 hours the mixture was neutralised using aqueous $NaHCO_3$ and the organic portion separated from the aqueous part. The aqueous layer was washed with $CHCl_3$ (3 × 10 mL) before all organic fractions were washed with brine (1 × 10 mL)dried ($MgSO_4$) and the solvent removed with a rotary evaporator to yield a yellow oil (0.65 g, 98%) requiring no further purification.

^{1}H NMR (400 MHz, $CDCl_3$): δ_H = 10.43 (s, 1H), 7.97 (dd, 1H, *J* = 9.0, 2.5), 7.87 (d, 1H, *J* = 2.5), 7.07 (d, 2H, *J* = 8.5), 6.98 (d, 2H, *J* = 85), 6.96 (d, 1H, *J* = 3.0), 6.82 (d, 2H, *J* = 8.5), 6.78 (d, 2H, *J* = 8.5), 6.73 (d, 1H, *J* = 9.0), 6.71 (d, 2H, *J* = 9.0), 6.61 (s, 1H), 6.54 – 6.60 (m, 1H), 6.28 (d, 1H, *J* = 3.0), 4.50 (s, 1H), 4.44 (s, 1H), 4.40 (s, 1H), 4.23 (s, 1H), 3.88 (d, 1H, *J* = 5.5), 3.86 (d, 1H, *J* = 5.5), 3.78 (d, 1H, *J* = 5.5), 1.62 – 1.82 (m, 3H), 1.16 – 1.58 (m, 27H), 1.02 – 1.15 (m, 36H), 0.82 – 1.01 (m, 18H);

^{13}C NMR (101 MHz, $CDCl_3$): δ_C = 190.1, 161.4, 155.0, 154.8, 153.7, 149.1, 142.9, 142.5, 141.2, 131.0, 130.7, 128.4, 128.0, 127.6, 126.4, 124.9, 124.0, 122.9, 120.1, 119.9, 113.6, 112.5, 112.2, 111.3, 110.1, 109.4, 71.4, 71.3, 70.7, 55.4, 53.8, 50.2, 49.2, 39.6, 39.5, 39.2, 30.7, 30.6, 30.5, 29.1, 29.0, 24.0, 23.9, 23.1, 23.0, 22.9, 17.9, 14.1, 12. 7, 12.6, 11.2, 11.1;

MS (ES+): m/z (%) = 1271 (100) [M + H^+], 1293 (15) [M + Na^+];

HRMS (ES+): calculated for $C_{77}H_{120}N_3O_8Si_2$ 1270.8614, found 1270.8586;

FT-IR (thin film): ν_{max}/cm^{-1} 2959, 2925, 2866, 1682, 1608, 1593, 1507, 1463, 1263, 1226, 1165, 1013.

Synthesis of **14**

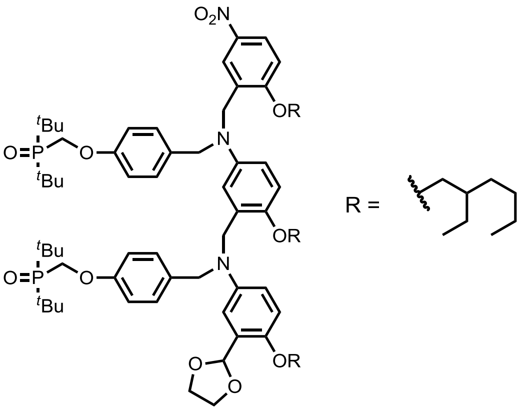

12’ (0.190 g, 0.24 mmol, 1 equiv.) and **10** (0.206 g, 0.36 mmol, 1.5 equiv.) were dissolved in DCE (900 μL) and $NaBH(OAc)_3$ (0.142 g, 0.67 mmol, 2.8 equiv.) was added with stirring. After 2 days the reaction was quenched with saturated aqueous $NaHCO_3$ solution, and extracted into $CHCl_3$ (4 × 10 mL). All the organic fractions were washed with water (1 × 10 mL), brine (1 × 10 mL) and dried ($MgSO_4$) before the solvent removed using a rotary evaporator. The crude product was purified using flash chromatography on silica eluting 0% to 10% MeOH in diethyl ether to yield a pale yellow oil (0.208 g, 65%).

^{1}H NMR (400 MHz, CD_3CN): δ_H = 7.98 (dd, 1H, *J* = 9.0, 3.0), 7.75 (d, 1H, *J* = 3.0), 7.10 (d, 2H, *J* = 9.0), 7.06 (d, 2H, *J* = 8.0), 6.87 – 6.97 (m, 5H), 6.74 (d, 1H, *J* = 9.0), 6.64 (d, 1H, *J* = 3.0), 6.58 (d, 1H, *J* = 9.0), 6.54 (dd, 1H, *J* = 9.0, 3.0), 6.28 – 6.35 (m, 2H), 5.93 (s, 1H), 4.48 (s, 2H), 4.45 (s, 2H), 4.40 (s, 2H), 4.34 (d, 2H, *J* = 3.0), 4.33 (d, 2H, *J* = 3.0), 4.17 (s, 2H), 3.96 (d, 2H, *J* = 5.5), 3.83 (br. d., 4H, *J* = 2.0), 3.72 – 3.78 (m, 4H), 1.55 – 1.78 (m, 3H), 1.16 – 1.53 (m, 60H), 0.78 – 0.95 (m, 18H);

^{13}C NMR (101 MHz, CD_3CN): δ_C = 162.8, 158.7 (d, *J* = 8.5), 158.6 (d, *J* = 8.5), 150.1, 143.6, 143.1, 142.0, 133.5, 133.4, 129.4, 129.2, 129.1, 128.2, 127.70, 125.2, 123.5, 115.4, 115.3, 114.7, 114.4, 114.1, 113.8, 112.7, 112.2, 100.0, 72.5, 72.4, 71.8, 65.8, 63.6 (d, *J* = 69.4), 63.5 (d, *J* = 70.1), 56.1, 55.1, 51.2, 50.6, 40.6, 40., 40.2, 36.0 (d, *J* = 57.8), 31.5, 31.4, 30.8, 29.9, 26.9, 24.9, 24.8, 23.9, 23.8, 14.6, 14.5, 11.7;

^{31}P NMR (162 MHz, CD_3CN): δ_P = 55.3, 55.3;

MS (ES+): m/z (%) = 676 (50) [M + 2H$^+$], 1351 (100) [M + H$^+$], 1373 (100) [M + Na$^+$], 1396 (40);

HRMS (ES+): calculated for $C_{79}H_{122}N_3O_{11}P_2$ 1350.8555, found 1350.8549;

FT-IR (thin film): ν_{max}/cm^{-1} 2958, 2927, 2871, 1680, 1610, 1592, 1505, 1465, 1432, 1392, 1264, 1225, 1174, 1135, 1044, 1017, 968.

Synthesis of **14’**

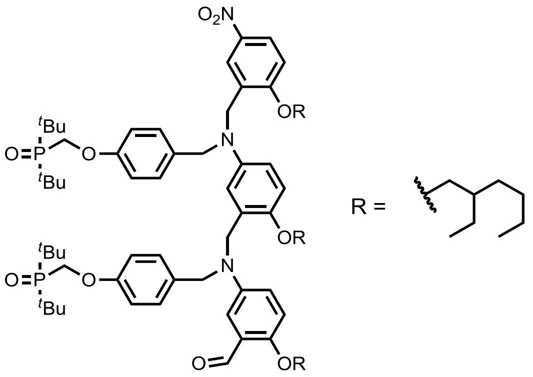

14 (0.21 g, 0.16 mmol, 1 equiv.) was dissolved in $CHCl_3$ (10 mL) and concentrated aqueous acid (10 mL) was added with stirring. After 3 days the mixture was neutralised using aqueous $NaHCO_3$ and the organic portion separated from the aqueous part. The aqueous layer was washed with $CHCl_3$ (3 × 10 mL) before all organic fractions were washed with brine (1 × 10 mL) dried ($MgSO_4$) and the solvent removed with a rotary evaporator to yield a yellow oil (0.20 g, 98%) requiring no further purification.

^{1}H NMR (400 MHz, $CDCl_3$): δ_H = 10.42 (s, 1H), 7.96 (d, 1H, *J* = 9.0), 7.84 (d, 1H, *J* = 3.0), 7.16 (d, 2H, *J* = 9.0), 7.06 (d, 2H, *J* = 9.0), 6.79 – 6.93 (m, 5H), 6.72 (dd, 2H, *J* = 9.0, 3.5), 6.64 (d, 1H, *J* = 9.5), 6.60 (dd, 1H, *J* = 9.0, 3.0), 6.51 – 6.57 (m, 1H), 6.26 (d, 1H, *J* = 3.0), 4.51 (s, 2H), 4.45 (s, 2H), 4.43 (s, 1H), 4.32 – 4.39 (m, 4H), 4.22 (s, 2H), 3.88 (d, 2H, *J* = 5.5), 3.86 (d, 2H, *J* = 5.5), 3.77 (d, 2H, *J* = 5.5), 1.75 (d, 2H, *J* = 7.0), 1.60 – 1.69 (m, 1H), 1.18 – 1.59 (m, 60H), 0.80 – 0.99 (m, 15H);

^{13}C NMR (101 MHz, $CDCl_3$): δ_C = 189.9, 161.3, 157.3 (d, *J* = 11.2), 157.2 (d, *J* = 10.8), 153.80, 149.10, 142.6, 142.1, 141.1, 132.1, 131.6, 128.1, 128.0, 127.7, 126.2, 124.8, 124.0, 122.7, 120.0, 114.1, 113.6, 112.4, 112.2, 111.3, 110.1, 109.2, 71.3, 71.2, 70.5, 62.6 (d, *J* = 70.1), 62.5 (d, *J* = 70.1), 55.3, 53.6, 50.3, 49.2, 39.5, 39.4, 39.1, 35.3 (d, *J* = 57.8), 30.5, 30.4, 30.3, 30.2, 29.0, 28.9, 26.4, 23.9, 23.8, 22.9, 22.8, 14.0, 13.9, 11.1;

^{31}P NMR (101 MHz, $CDCl_3$): δ_P = 56.8;

FT-IR (thin film): ν_{max}/cm^{-1} 2958, 2928, 2872, 1683, 1592, 1508, 1466, 1339, 1265, 1229, 1176, 1138, 1081, 1047, 1014, 968.

Synthesis of **15**

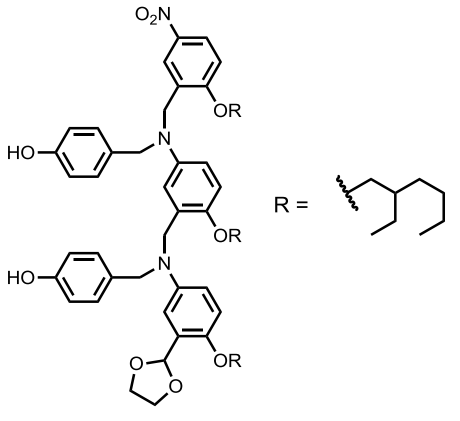

13 (0.321 g, 0.24 mmol, 1 equiv.) was dissolved in THF (2 mL) at 0 °C and TBAF (720 μL, 0.72 mmol, 3 equiv.) was added with stirring. After 1 hour water (5 mL) was added and the aqueous mixture washed with diethyl ether (4 × 10 mL). All organic fractions were combined and washed with brine (1 × 10 mL) dried ($MgSO_4$) and the solvent removed with a rotary evaporator. The crude mixture was then purified via flash chromatography on silica eluting with a gradient from 20% to 60% of diethyl ether in hexane to yield a light red oil (0.189 g, 77%).

^{1}H NMR (400 MHz, $CDCl_3$): δ_H = 8.09 (dd, 1H, *J* = 9.0, 2.5), 8.01 (d, 1H, *J* = 2.5), 7.05 (d, 2H, *J* = 8.5), 6.93 (d, 2H, *J* = 8.5), 6.87 (d, 1H, *J* = 9.0), 6.61 – 6.77 (m, 4H), 6.51 – 6.60 (m, 3H), 6.37 – 6.46 (m, 2H), 6.28 (dd, 1H, *J* = 9.0, 3.0), 6.09 (s, 1H), 4.54 (s, 2H), 4.44 (s, 2H), 4.41 (s, 2H), 4.29 (s, 2H), 3.91 – 4.06 (m, 6H), 3.80 (d, 2H, *J* = 6.0), 3.75 (d, 2H, *J* = 5.5), 1.57 – 1.82 (m, 3H), 1.18 – 1.56 (m, 24H), 0.80 – 0.98 (m, 18H);

^{13}C NMR (126 MHz, $CDCl_3$): δ_C = 161.7, 154.6, 154.4, 149.0, 143.1, 142.5, 141.3, 131.3, 130.5, 128.7, 128.1, 127.7, 127.4, 125.5, 124.3, 123.1, 115.8, 115.3, 113.7, 113.6, 113.5, 112.1, 111.3, 111.1, 110.4, 100.3, 71.8, 71.3, 70.7, 65.9, 65.0, 56.0, 54.5, 50.9, 49.7, 39.6, 39.5, 39.2, 30.6, 30.5, 29.1, 24.0, 23.9, 23.1, 23.0, 22.9, 14.1, 14.0, 11.1;

MS (ES+): m/z (%) = 853 (10), 897 (35), 959 (10), 1003 (100) [M + H^+], 1025 (25);

HRMS (ES+): calculated for $C_{61}H_{84}N_3O_6$ 1002.6208, found 1002.6224;

FT-IR (thin film): ν_{max}/cm^{-1} 2957, 2927, 2871, 1613, 1593, 1505, 1462, 1337, 1261, 1223, 1167, 1077, 1015, 964.

Synthesis of **16**

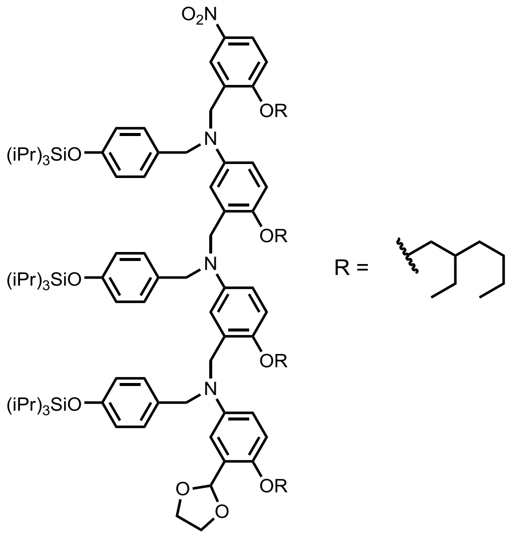

13' (0.885 g, 0.70 mmol, 1 equiv.) and **9** (0.774 g, 1.4, mmol, 2 equiv.) were dissolved in DCE (0.5 mL) and $NaBH(OAc)_3$ (0.413 g, 2.0 mmol, 2.8 equiv.) was added with stirring. After 2 days the reaction was quenched with saturated aqueous $NaHCO_3$ solution, and extracted into $CHCl_3$ (4 × 10 mL). All the organic fractions were washed with water (1 × 10 mL), brine (1 × 10 mL) and dried ($MgSO_4$) before the solvent removed on a rotary evaporator. The crude product was purified using flash chromatography on silica eluting with a gradient from 0% to 5% of diethyl ether in hexane to yield a golden yellow oil (1.00 g, 79%).

^{1}H NMR (500 MHz, $CDCl_3$): δ_H = 7.98 – 8.02 (m, 2H), 7.05 (d, 2H, *J* = 8.5), 7.00 (d, 2H, *J* = 8.5), 6.96 (d, 2H, *J* = 8.5), 6.89 (d, 1H, *J* = 3.0), 6.82 (d, 2H, *J* = 8.5), 6.79 (d, 2H, *J* = 8.5), 6.77 (d, 2H, *J* = 8.5), 6.72 (d, 1H, *J* = 4.0), 6.71 (d, 1H, *J* = 4.0), 6.69 (d, 1H, *J* = 9.0), 6.59 (d, 1H, *J* = 9.0), 6.51 – 6.55 (m, 2H), 6.49 (d, 1H, *J* = 2.9), 6.44 (d, 1H, *J* = 3.0), 6.27 – 6.30 (m, 1H), 6.18 (s, 1H), 4.50 (s, 2H), 4.48 (s, 2H), 4.46 (s, 2H), 4.44 (s, 2H), 4.10 (s, 2H), 4.07 (s, 2H), 3.92 (s, 4H), 3.79 – 3.83 (m, 6H), 3.77 (d, 2H, *J* = 5.5), 1.72 (d, 4H, *J* = 6.2), 1.21 – 1.57 (m, 35H), 1.04 – 1.18 (m, 54H), 0.86 – 0.99 (m, 24H);

^{13}C NMR (63 MHz, $CDCl_3$): δ_C = 161.4, 154.9, 154.5, 149.0, 148.9, 148.4, 143.3, 143.1, 142.6, 141.3, 131.8, 131.6, 131.7, 128.7, 128.2, 127.7, 127.6, 127.0, 126.8, 124.1, 123.2, 119.8, 119.7, 119.6, 114.2, 113.6, 113.3, 112.5, 112.2, 112.1, 111.6, 111.4, 110.9, 110.3, 99.8, 71.9, 71.3, 70.8, 70.7, 64.9, 55.4, 54.3, 53.7, 50.5, 50.2, 49.7, 39.6, 39.5, 39.2, 30.7, 30.6, 30.5, 29.1, 24.0, 23.9, 23.1, 23.0, 17.9, 14.1, 14.0, 12.6, 11.2, 11.1;

MS (ES+): m/z (%) = 1810 (80) [M + H^+], 1811 (100), 1812 (70), 1813 (40);

HRMS (ES+): calculated for $C_{110}H_{173}N_4O_{11}Si_3$ 1810.2409, found 1810.2484;

FT-IR (thin film): ν_{max}/cm^{-1} 2959, 2929, 2867, 1608, 1508, 1465, 1340, 1264, 914.

Synthesis of **16'**

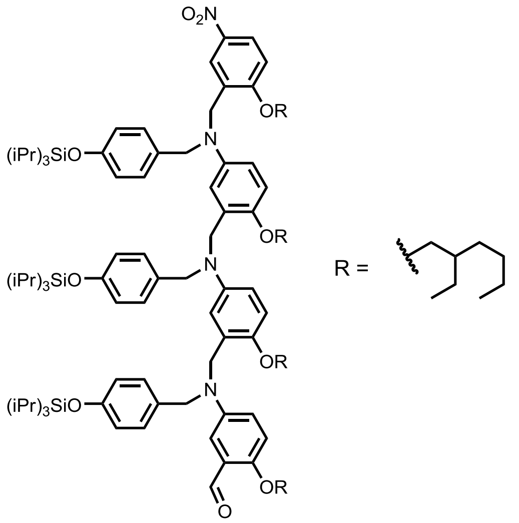

16 (0.89 g, 0.49 mmol, 1 equiv.) was dissolved in $CHCl_3$ (10 mL) and concentrated aqueous acid (10 mL) was added with stirring. After 2 days the mixture was neutralised using aqueous $NaHCO_3$ and the organic portion separated from the aqueous part. The aqueous layer was washed with $CHCl_3$ (3 × 10 mL) before all organic fractions were washed with brine (1 × 10 mL) dried ($MgSO_4$) and the solvent removed using a rotary evaporator to yield a yellow oil (0.78 g, 90%) requiring no further purification.

^{1}H NMR (500 MHz, $CDCl_3$): δ_H = 10.46 (s, 1H), 7.99 – 8.03 (m, 2H), 7.14 (d, 1H, *J* = 2.0), 7.03 (d, 2H, *J* = 8.5), 6.97 (d, 2H, *J* = 8.5), 6.92 (d, 2H, *J* = 8.5), 6.72 – 6.80 (m, 9H), 6.67 (d, 1H, *J* = 8.5), 6.59 (d, 1H, *J* = 8.5), 6.50 (dd, 1H, *J* = 8.5, 3.0), 6.46 (d, 1H, *J* = 3.0), 6.34 (d, 1H, *J* = 3.0), 6.30 (dd, 1H, *J* = 9.0, 3.0), 4.48 (s, 2H), 4.45 (s, 2H), 4.42 (s, 4H), 4.10 (s, 2H), 4.07 (s, 2H), 3.85 (d, 4H, *J* = 5.5), 3.79 (d, 2H, *J* = 6.0), 3.72 (d, 2H, *J* = 6.0), 1.67 – 1.77 (m, 3H), 1.57 – 1.67 (m, 2H), 1.18 – 1.56 (m, 41H), 1.04 – 1.16 (m, 54H), 0.84 – 0.96 (m, 24H);

^{13}C NMR (63 MHz, $CDCl_3$): δ_C = 189.9, 161.4, 154.9, 154. 7, 154.6, 153.7, 149.1, 148.5, 143.4, 143.0, 142.7, 141.3, 131.7, 131.1, 131.0, 128.8, 128.2, 127.7, 127.6, 127.5, 126.2, 125.1, 124.0, 123.2, 120.7, 119.8, 119.7, 113.9, 113.2, 112.3, 112.2, 112.1, 111.4, 111.1, 110.3, 110.1, 71.4, 71.3, 70.8, 70.7, 55.4, 53.4, 50.5, 50.3, 49.6, 39.6, 39.5, 39.2, 30.7, 30.6, 30.5, 29.1, 29.0, 24.0, 23.9, 23.1, 23.0, 22.9, 17.9, 14.1, 14.0, 12.7, 12.6, 11.2, 11.1;

MS (ES+): m/z (%) = 1765 (40), 1766 (100) [M + H^+], 1767 (95), 1768 (50), 1769 (20);

HRMS (ES+): calculated for $C_{108}H_{169}N_4O_{10}Si_3$ 1766.2147, found 1766.2076;

FT-IR (thin film): ν_{max}/cm^{-1} 2959, 2929, 2867, 1683, 1608, 1507, 1464, 1340, 1264, 913.

Synthesis of **17**

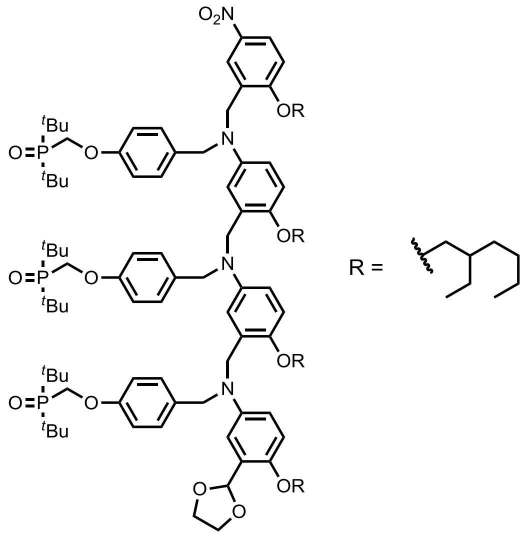

14' (0.211 g, 0.16 mmol, 1 equiv.) and **10** (0.185 g, 0.32 mmol, 2.0 equiv.) were dissolved in DCE (1100 μL) and $NaBH(OAc)_3$ (0.096 g, 0.45 mmol, 2.8 equiv.) was added with stirring. After 4 days the reaction was quenched with saturated aqueous $NaHCO_3$ solution, and extracted into $CHCl_3$ (4 × 10 mL). All the organic fractions were washed with water (1 × 10 mL), brine (1 × 10 mL) and dried ($MgSO_4$) before the solvent removed with a rotary evaporator. The crude product was purified using flash chromatography on silica eluting with a gradient from 0% to 10% MeOH in EtOAc to yield a pale yellow oil (0.093 g, 31%).

^{1}H NMR (400 MHz, $CDCl_3$): δ_H = 7.98 (dd, 1H, *J* = 9.0, 3.0), 7.94 (d, 1H, *J* = 2.5), 7.09 (d, 2H, *J* = 8.5), 7.02 (d, 2H, *J* = 8.5), 6.97 (d, 2H, *J* = 8.5), 6.87 (d, 1H, *J* = 3.0), 6.71 – 6.84 (m, 7H), 6.64 – 6.71 (m, 2H), (d, 1H, *J* = 9.0), 6.43 – 6.50 (m, 3H), 6.40 (d, 1H, *J* = 2.5), (dd, 1H, *J* = 8.5, 2.5), 6.10 (s, 1H), 4.42 (br. s., 8H), 4.30 – 4.37 (m, 6H), 4.03 (br. s., 4H), 3.92 (d, 4H, *J* = 3.3), 3.83 (d, 2H, *J* = 5.5), 3.70 – 3.78 (m, 6H), 1.57 – 1.75 (m, 4H), 1.16 – 1.53 (m, 86H), 0.77 – 0.94 (m, 24H);

^{13}C NMR (101 MHz, $CDCl_3$): δ_C = 161.3, 157.2, 157.1, (d, *J* = 10.8), 149.1, 149.0, 148.5, 143.1, 142.8, 142.2, 141.1, 132.8, 132.5, 132.1, 128.4, 128.3, 128.0, 127.3, 126.7, 126.6, 124.0, 123.0, 114.0, 113.9, 113.8, 113.6, 113.3, 112.5, 112.1, 111.6, 111.4, 110.9, 110.2, 99.5, 71.8, 71.2, 70.7, 70.6, 64.8, 62.5 (d, *J* = 71), 55.1, 54.1, 53.3, 50.3, 49.4, 39.4, 39.3, 39.0, 35.2, 35.3 (d, *J* = 58.0), 30.5, 30.4, 30.3, 28.9, 23.8, 23.7, 22.9, 22.8, 14.0, 13.9, 11.0;

^{31}P NMR (162 MHz, $CDCl_3$): δ_P = 56.6, 56.6, 56.5;

MS (ES+): m/z (%) = 1862 (50), 1863 (100) [M + H$^+$], 1864 (100), 1865 (50), 1866 (20);

FT-IR (thin film): ν_{max}/cm^{-1} 2957, 2926, 2871, 1610, 1591, 1506, 1467, 1339, 1265, 1225, 1142, 965.

Synthesis of **17'**

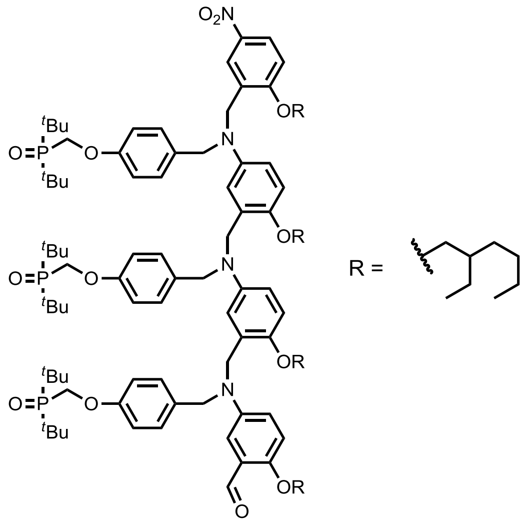

17 (0.093 g, 0.051 mmol, 1 equiv.) was dissolved in $CHCl_3$ (5 mL) and concentrated aqueous acid (5 mL) was added with stirring. After 2 days the mixture was neutralised using aqueous $NaHCO_3$ and the organic portion separated from the aqueous part. The aqueous layer was washed with $CHCl_3$ (3 × 10 mL) before all organic fractions were washed with brine (1 × 10 mL) dried ($MgSO_4$) and the solvent removed with a rotary evaporator to yield a yellow oil (0.082 g, 88%) requiring no further purification.

^{1}H NMR (400 MHz, $CDCl_3$): δ_H = 10.42 (s, 1H), 8.01 (dd, 1H, *J* = 9.0, 2.5), 7.94 (d, 1H, *J* = 2.5), 7.10 (d, 2H, *J* = 8.5), 6.95 – 7.07 (m, 5H), 6.76 – 6.83 (m, 7H), 6.73 (d, 2H, *J* = 1.0), 6.65 (d, 1H, *J* = 9.0), 6.60 (d, 1H, *J* = 9.0), 6.43 – 6.50 (m, 2H), 6.28 – 6.34 (m, 2H), 4.41 – 4.47 (m, 6H), 4.30 – 4.40 (m, 9H), 4.08 (br. s., 4H), 3.88 (d, 2H, *J* = 5.5), 3.83 (d, 2H, *J* = 5.5), 3.77 (d, 2H, *J* = 5.5), 3.67 (d, 2H, *J* = 5.5), 1.63 – 1.78 (m, 3H), 1.53 – 1.63 (m, 1H), 1.17 – 1.52 (m, 86H), 0.78 – 0.95 (m, 24H);

^{13}C NMR (101 MHz, $CDCl_3$): δ_C = 189.8, 161.4, 157.3 (d, *J* = 10.8), 157.1 (d, *J* = 11.5), 157.0 (d, *J* = 11.0), 153.8 ,149.2, 148.6, 143.2, 142.7, 142.3, 141.2, 132.7, 132.2, 131.8, 128.5, 128.4, 127.9, 127.8, 127.2, 126.0, 125.1, 124.1, 123.1, 120.4, 114.0, 113.9, 113.3, 112.4, 112.2, 112.1, 111.5, 111.2, 110.2, 110.1, 71.4, 71.3, 70.8, 70.7, 62.6 (d, *J* = 70.1), 62.5 (d, *J* = 70.1), 55.3, 54.4, 53.2, 50.4, 50.3, 49.5, 39.5, 39.4, 39.1, 35.4 (d, *J* = 57.8), 30.6, 30.4, 29.6, 29.0, 28.9, 26.4, 23.9, 23.8, 23.0, 22.9, 22.8, 14.0, 11.1, 11.0;

^{31}P NMR (162 MHz, $CDCl_3$): δ_P = 56.8, 56.7, 56.6;

MS (ES+): m/z (%) = 1820 (80) [M + H^+], 1821 (100), 1842 (80) [M + Na^+], 1843 (100);

HRMS (ES+): calculated for $C_{108}H_{166}N_4O_{13}P_3$ 1820.1664, found 1820.1619;

FT-IR (thin film): ν_{max}/cm^{-1} 2957, 2921, 2852, 1683, 1508, 1466, 1233, 1138.

Synthesis of **18**

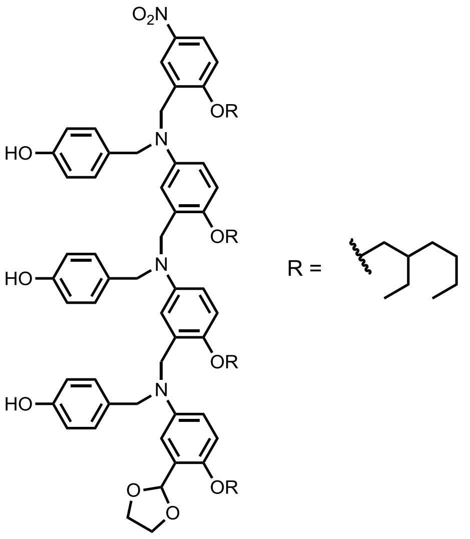

16 (0.122 g, 0.07 mmol, 1 equiv.) was dissolved in THF (2 mL) at 0 °C and TBAF (210 μL, 0.21 mmol, 3 equiv.) was added with stirring. After 1 hour water (5 mL) was added and the aqueous mixture washed with diethyl ether (3 × 10 mL). All organic fractions were combined and washed with brine (1 × 5 mL) dried ($MgSO_4$) and the solvent removed with a rotary evaporator. The crude mixture was then purified via flash chromatography on silica eluting with a gradient from 20% to 70% of diethyl ether in hexane to yield a light red oil (0.087 g, 96%).

^{1}H NMR (500 MHz, $CDCl_3$): δ_H = 8.11 (dd, 1H, *J* = 9.0, 3.0), 8.04 (d, 1H, *J* = 2.5), 6.98 (d, 2H, *J* = 8.5), 6.85 – 6.89 (m, 3H), 6.83 (d, 2H, *J* = 8.5), 6.81 (d, 1H, *J* = 3.5), 6.66 – 6.72 (m, 5H), 6.60 (d, 2H, *J* = 8.5), 6.47 – 6.51 (m, 2H), 6.37 – 6.45 (m, 4H), 6.34 (d, 1H, *J* = 3.0), 6.14 (s, 1H), 4.58 (s, 2H), 4.48 (s, 2H), 4.45 (s, 2H), 4.38 (s, 2H), 4.09 (s, 2H), 3.98 – 4.08 (m, 4H), 3.96 (d, 2H, *J* = 5.7), 3.87 (s, 2H), 3.81 (d, 2H, *J* = 5.7), 3.73 – 3.77 (m, 4H), 1.74 – 1.81 (m, 1H), 1.62 – 1.74 (m, 3H), 1.22 – 1.54 (m, 32H), 0.84 – 0.97 (m, 24H);

^{13}C NMR (126 MHz, $CDCl_3$): δ_C = 161.7, 154.7, 154.2, 154.1, 149.2, 149.0, 148.1, 143.4, 143.2, 142.3, 141.4, 131.6, 131.4, 130.5, 129.0, 128.1, 127.9, 127.5, 127.4, 126.9, 125.2, 124.3, 123.2, 115.6, 115.5, 115.3, 114.3, 113.8, 113.3, 112.6, 112.3, 112.0, 111.8, 110.8, 110.7, 110.3, 100.4, 71.7, 71.4, 71.0, 70.6, 65.0, 55.6, 54.9, 54.2, 51.3, 50.8, 49.5, 39.7, 39.5, 39.2, 30.7, 30.5, 29.1, 29.0, 24.0, 23.9, 23.8, 23.1, 23.0, 22.9, 14.1, 14.0, 11.2, 11.1, 11.0;

MS (ES+): m/z (%) = 565 (30), 618 (75), 671 (75), 1342 (100) [M + H^+];

HRMS (ES+): calculated for $C_{83}H_{113}N_4O_{11}$ 1341.8406, found 1341.8342;

FT-IR (thin film): ν_{max}/cm^{-1} 2956, 2918, 2850, 2161, 2030, 1616, 1504, 1338, 1261, 1022.

Synthesis of **19**

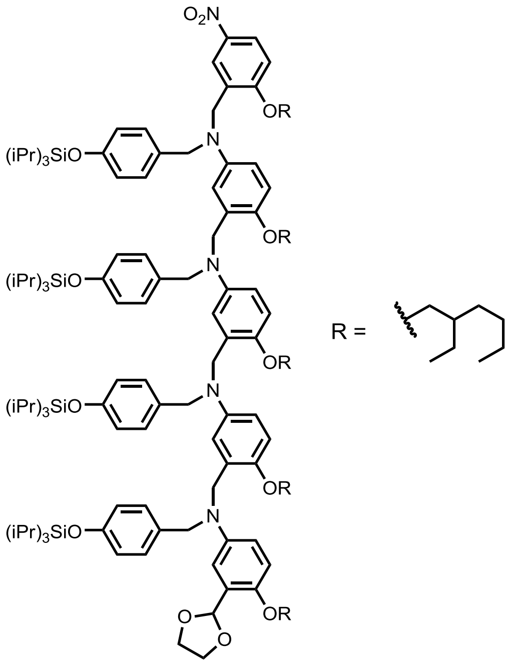

16' (0.815 g, 0.06 mmol, 1 equiv.) and **9** (0.713 g, 0.13 mmol, 2 equiv.) were dissolved in DCE (0.5 mL) and $NaBH(OAc)_3$ (0.038 g, 0.13 mmol, 2.8 equiv.) was added with stirring. After 2 days the reaction was quenched with saturated aqueous $NaHCO_3$ solution, and extracted into $CHCl_3$ (4 × 10 mL). All the organic fractions were washed with water (1 × 10 mL), brine (1 × 10 mL) and dried ($MgSO_4$) before the solvent removed with a rotary evaporator. The crude product was purified using flash chromatography on silica eluting with a gradient from 3% to 5% of diethyl ether in hexane to yield a golden yellow oil (0.036 g, 26%).

^{1}H NMR (400 MHz, CDCl$_3$): δ_H = 7.96 – 8.01 (m, 2H), 7.03 (d, 2H, *J* = 8.5), 6.97 (d, 2H, *J* = 8.5), 6.89 (d, 4H, *J* = 8.5), 6.64 – 6.81 (m, 11H), 6.60 – 6.64 (m, 2H), 6.46 – 6.57 (m, 6H), 6.43 (dd, 1H, *J* = 9.0, 2.5), 6.30 (dd, 1H, *J* = 9.0, 3.0), 6.15 (s, 1H), 4.45 (s, 2H), 4.42 (s, 2H), 4.40 (s, 4H), 4.38 (s, 2H), 4.10 (s, 2H), 4.06 (s, 2H), 4.04 (s, 2H), 3.79 – 3.87 (m, 6H), 3.70 – 3.77 (m, 8H), 1.58 – 1.75 (m, 5H), 1.15 – 1.54 (m, 52H), 0.99 – 1.15 (m, 72H), 0.81 – 0.96 (m, 30H);

^{13}C NMR (101 MHz, CDCl$_3$): δ_C = 161.4, 154.9, 154.5, 154.4, 149.2, 148.9, 148.6, 148.5, 143.4, 143.3, 142.8, 141.3, 132.1, 131.7, 131.1, 128.8, 128.3, 127.9, 127.8, 127.7, 127.3, 126.9, 126.8, 125.5, 124.0, 123.3, 119.8, 119.7, 119.6, 114.0, 113.6, 113.5, 113.3, 113.1, 112.3, 112.2, 111.8, 111.6, 111.5, 111.4, 110.1, 99.9, 71.8, 71.3, 70.9, 70.7, 64.8, 55.4, 54.5, 54.3, 53.6, 50.9, 50.4, 50.0, 49.9, 39.6, 39.5, 39.1, 30.6, 30.5, 30.3, 29.2, 29.1, 29.0, 24.0, 24.9, 23.9, 23.1, 23.0, 17.9, 14.1, 14.0, 12.6, 11.2, 11.1;

MS (ES+): m/z (%) = 2305 (50), 2306 (100) [M + H^+], 2307 (95), 2308 (55), 2309 (20);

FT-IR (thin film): ν_{max}/cm^{-1} 2957, 2927, 2859, 1680, 1610, 1592, 1500, 1464, 1394, 1338, 1265, 1249, 1231, 1170, 1081, 1013, 970.

Synthesis of **20**

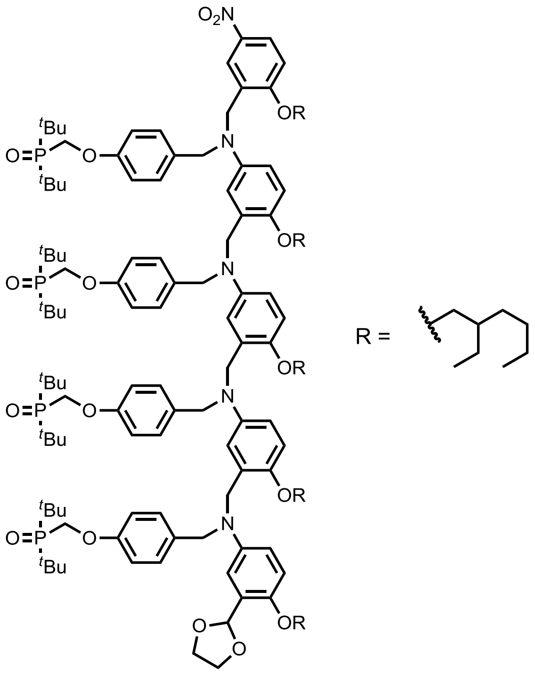

17' (0.106 g, 0.06 mmol, 1 equiv.) and **10** (0.12 g, 0.21 mmol, 3.5 equiv.) were dissolved in DCE (0.5 mL) and $NaBH(OAc)_3$ (0.035 g, 0.16 mmol, 2.8 equiv.) was added with stirring. After 1 week, more $NaBH(OAc)_3$ (0.035 g, 0.16 mmol, 2.8 equiv.) was added to the reaction. After another week the reaction was quenched with saturated aqueous $NaHCO_3$ solution, and extracted into $CHCl_3$ (4 × 10 mL). All the organic fractions were washed with water (1 × 10 mL), brine (1 × 10 mL) and dried ($MgSO_4$) before the solvent removed with a rotary evaporator. The crude product was purified using flash chromatography on silica eluting with a gradient from 0% to 10% of MeOH in a 25:75 mixture of $CHCl_3$/EtOAc and then 10% MeOH in neat $CHCl_3$ to yield a golden yellow oil (0.021 g, 15%).

^{1}H NMR (400 MHz, $CDCl_3$): δ_H = 7.99 (dd, 1H, *J* = 9.0, 2.5), 7.93 (d, 1H, *J* = 2.5), 7.11 (d, 1H, *J* = 8.5), 7.02 (d, 1H, *J* = 8.5), 6.96 (d, 1H, *J* = 8.5), 6.92 (d, 1H, *J* = 8.5), 6.70 – 6.85 (m, 9H), 6.64 – 6.69 (m, 2H), 6.57 – 6.63 (m, 2H), 6.55 (d, 1H, *J* = 2.5), 6.44 – 6.51 (m, 4H), 6.41 (dd, 1H, *J* = 9.0, 3.0), 6.33 (dd, 1H, *J* = 9.0, 3.0), 6.10 (s, 1H), 4.40 (br. s., 9H), 4.29 – 4.36 (m, 9H), 4.11 (s, 2H), 4.01 (d, 4H, *J* = 5.0), 3.90 (s, 4H), 3.85 (d, 2H, *J* = 5.5), 3.69 – 3.77 (m, 8H), 1.56 – 1.76 (m, 5H), 1.18 – 1.49 (m, 112H), 0.79 – 0.93 (m, 30H);

^{13}C NMR (101 MHz, $CDCl_3$): δ_C = 161.4, 157.3 (d, *J* = 11.0), 157.0 (d, *J* = 11.0), 156.9 (d, *J* = 11.0), 149.4, 149.2, 148.7, 143.3, 143.2, 143.1, 142.5, 141.3, 133.1, 132.7, 132.6, 132.2, 128.5, 128.2, 128.1, 127.6, 127.1, 126.7, 124.1, 123.3, 114.1, 113.9, 113.7, 113.6, 113.3, 113.2, 112.4, 112.3, 111.8, 111.7, 111.6, 110.2, 99.7, 72.0, 71.4, 71.0, 70.9, 70.8, 65.0, 62.6 (d, *J* = 71.0), 55.3, 54.4, 54.1, 53.2, 50.8, 50.3, 50.1, 49.5, 39.5, 39.4, 39.1, 35.4 (d, *J* = 58.0), 30.6, 30.5, 30.4, 29.7, 29.1, 29.0, 26.4, 23.9, 23.8, 23.0, 22.9, 14.1, 14.0, 11.2, 11.1;

^{31}P NMR (162 MHz, $CDCl_3$): δ_P = 56.8, 56.7;

MS (ES+): m/z (%) = 2379 (100), 2395 (60), 2378 (30) [M + H$^+$], 2379 (50), 2395 (30);

FT-IR (thin film): ν_{max}/cm^{-1} 2958, 2920, 2851, 1659, 1632, 1506, 1470, 1427, 1243, 1137.

Synthesis of **21**

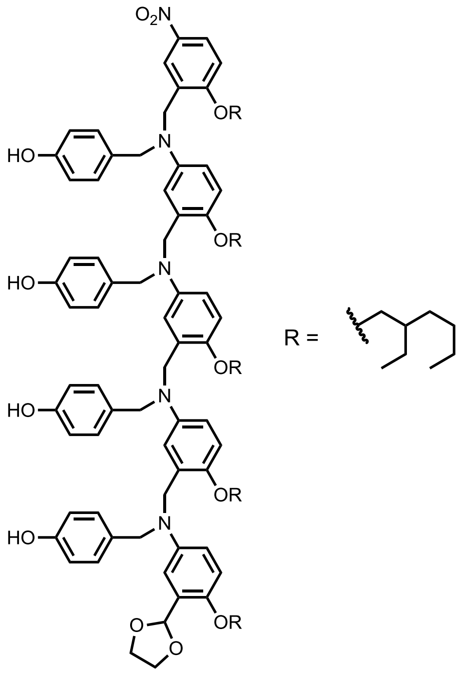

19 (0.690 g, 0.30 mmol, 1 equiv.) was dissolved in THF (10 mL) at 0 °C and TBAF (2390 μL, 2.4 mmol, 8 equiv.) was added with stirring. After 1 hour water (10 mL) was added and the aqueous mixture washed with diethyl ether (3 × 10 mL). All organic fractions were combined and washed with brine (1 × 10 mL) dried ($MgSO_4$) and the solvent removed with a rotary evaporator. The crude mixture was then purified via flash chromatography on silica eluting with a gradient from 30% to 80% of diethyl ether in hexane to yield a light red oil (0.105 g, 20%).

^{1}H NMR (400 MHz, $CDCl_3$): δ_H = 8.10 (dd, 1H, *J* = 9.0, 2.5), 8.04 (d, 1H, *J* = 2.5), 6.90 – 6.99 (m, 4H), 6.80 – 6.89 (m, 4H), 6.76 (d, 2H, *J* = 8.5), 6.66 – 6.73 (m, 6H), 6.56 – 6.64 (m, 5H), 6.53 (dd, 1H, *J* = 9.0, 3.0), 6.33 – 6.46 (m, 7H), 6.17 (s, 1H), 4.45 – 4.56 (m, 8H), 4.41 (s, 2H), 3.93 – 4.10 (m, 10H), 3.75 – 3.84 (m, 10H), 1.63 – 1.82 (m, 5H), 1.23 – 1.56 (m, 40H), 0.84 – 0.97 (m, 30H);

^{13}C NMR (101 MHz, $CDCl_3$): δ_C = 161.7, 154.5, 154.3, 154.2, 154.1, 149.2, 149.0, 148.1, 148.0, 143.6, 143.3, 143.1, 142.5, 141.4, 131.4, 131.2, 130.7, 128.8, 128.1, 128.0, 127.7, 127.6, 127.2, 126.9, 125.2, 124.3, 123.2, 115.6, 115.5, 115.3, 114.4, 113.9, 113.5, 112.9, 112.6, 112.3, 112.1, 111.8, 111.1, 110.8, 110.7, 110.3, 100.3, 71.8, 71.4, 71.3, 71.0, 70.7, 65.0, 55.4, 54.8, 54.3, 51.3, 50.8, 50.6, 49.6, 39.6, 39.5, 39.2, 30.7, 30.6, 30.5, 30.4, 29.1, 29.0, 24.0, 23.9, 23.8, 23.0, 22.9, 14.0, 11.2, 11.1, 11.0;

MS (ES+): m/z (%) = 484 (100), 1575 (10), 1680 (10), 1681 (50) [M + H^+], 1682 (50), 1683 (25), 1684 (10);

HRMS (ES+): calculated for $C_{105}H_{142}N_5O_{13}$ 1681.0604, found 1681.0530;

FT-IR (thin film): ν_{max}/cm^{-1} 2957, 2927, 2858, 2163, 1613, 1593, 1505, 1464, 1339, 1264, 1225, 1168.

Section S4: References

1 M. A. Ischay, Z. Lu, and T. P. Yoon, *J. Am. Chem. Soc.*, 2010, **132**, 8572–8574.

2 H. Sun, C. A. Hunter, C. Navarro, and S. Turega, *J. Am. Chem. Soc.*, 2013, **135**, 13129–13141.
